# Education, relationships, and place: life choices in the narratives of university master students

**DOI:** 10.3389/fpsyg.2024.1232370

**Published:** 2024-04-17

**Authors:** Oxana Mikhaylova, Alexandra Bochaver, Victoria Yerofeyeva

**Affiliations:** ^1^Centre for Modern Childhood Research, HSE University, Moscow, Russia; ^2^Department for Social Institutions Analysis, HSE University, Moscow, Russia

**Keywords:** life choices, fortunate choices, unfortunate choices, narrative analysis, McAdams, essays, written communication, university students

## Abstract

Choice is one of the most roughly defined concepts in contemporary social sciences. Previous studies have elucidated the factors that influence young people’s choices in different life situations. However, it is still unclear how young people evaluate these choices and how they integrate them into their biographies. In this study, we examine the narratives of 30 first-year master’s students at HSE University with regard to two categories of life choices: those that they perceive as fortunate and those that they perceive as unfortunate. Using a written online survey, the data was collected in the spring of 2022. To categorize the different decision kinds, thematic analysis was applied. Overall, we discovered that narratives about the life choices made by master students concentrated on education, relationships and place.

## 1 Introduction

Choice is a vague concept in contemporary social sciences. Firstly, choice can be equated with decision-making, and secondly, choice can be considered a turning point in a life story. A significant part of psychological research is devoted to the study of preferences from a limited number of alternatives, personality traits, and cognitive distortions/biases (Kahneman, 2003; Galotti, 2007; Leontiev et al., 2020).

Structural, cultural, interpersonal, and personal factors also influence young people’s choices (Eun et al., 2013; Guan et al., 2015; Mitra and Arnett, 2021). The number of available options is one of the *structural* factors that could make the process of choosing harder for people (Schwartz et al., 2002). Having many alternatives can lead to regret due to the fact that it is impossible to try them all. Additionally, personal autonomy influences choice; specifically, when a person feels more autonomous, it is easier for them to make a choice coherent with their values (Ryan and Deci, 2006). For example, students that can better self-regulate during decision-making are more likely to choose a professional trajectory that corresponds with their specialization in college (Eun et al., 2013). Concurrently, the existence of options increases motivation, life satisfaction, and wellbeing (Bone et al., 2014). Therefore, when people make choices based on their intrinsic motivation, they have more successful educational experiences, feel more satisfied with their job, and progress more quickly up the career ladder (Ryan and Deci, 2000). Values are one of the *cultural* factors that influence decision-making. Young people in countries with a collectivist type of culture take into account social expectations when making choices related to their education and career, while young people from an individualistic type of culture tend to pay less attention to others’ opinions (Mitra and Arnett, 2021). If a country has both collectivist and individualistic values, young people balance their own thoughts and wishes about their future with social expectations (Akosah-Twumasi et al., 2018). *Confidants’ opinions* are a significant interpersonal factor that can impact young people’s choices (Sanfey, 2007). For instance, students may choose an educational path that they do not like personally due to being strongly influenced by important members of their social circles, such as parents, teachers, and significant others (Guan et al., 2015). Self-esteem, self-efficacy, and personal interests are *personal factors* that are important in choice (Galotti et al., 2006; Parker et al., 2012; Skatova and Ferguson, 2014). Moreover, Kunnen (2013) identified that young people tend to consider educational choices as fortunate if they correspond with individual preferences, improve their wellbeing and job satisfaction, and make them feel more stable. Furthermore, age-related factors can affect choice among different age groups. For example, adolescents tend to postpone life choices (Bochaver, 2008), but young people tend to make more choices than people of other ages. During youth, a lot of people tend to make career decisions and other important decisions that could determine their future life (Gati and Saka, 2001; Mather, 2006; Reed et al., 2008). These choices are developmental tasks that help young people become more responsible for their lives and the lives of their significant others (Schulenberg et al., 2004; Mayseless and Keren, 2014; Arnett, 2015).

### 1.1 Narrative identity and the promise of narrative analysis framework for life choices investigations

Overall, previous studies have shown how young people make choices, but it is unclear how they evaluate their choices in later life and in relation to their biography. To address this research question, we used the concept of narrative identity (McAdams, 2011). Reflection on personal life in the forms of internal and external monologues is an important part of the individual process of aging (McAdams and Pals, 2006; McAdams, 2011). By thinking about personal life experiences and evaluating them, individuals may alter their future (Kaźmierczak et al., 2021). Concurrently, self-life reflections constitute narrative identity and represent an important life path development factor (McAdams, 2011). To be precise, narrative identity is a life story that is based on individual experiences, their evaluations, values, life goals, and visions of the future. Studies on narrative identity building in relation to different life spheres and at various life stages have helped scholars to identify how psychological disorders and wellbeing manifest themselves and may be identified through personal self-representations in daily life (Cowan et al., 2021; Shiner et al., 2021). Additionally, narratives are important for counseling, since they provide material for professional coaches, psychologists, and other mental health specialists to guide critical self-improvement sessions with clients and help them to build more meaningful and prosperous lives (Newitt et al., 2019; Singer, 2019; Turner et al., 2021).

### 1.2 Study rationale

In this paper, we focus on the analysis of university students’ narrative identity building in relation to two types of life choices: choices that they consider fortunate and choices that they consider unfortunate. In this work, choices refer to independent decisions based on intrinsic motivation (Ryan and Deci, 2000) that a person makes during the current period of their life and that determine their life trajectory (Arnett, 2000). Our goal is to compare the narratives on fortunate and unfortunate choices to reveal what types of choices are considered fortunate and what types are thought to be unfortunate. In terms of studying fortunate and unfortunate choices, we base the classification of choices as fortunate or unfortunate on individual perceptions and do not evaluate the meaning of the choices for the participants’ biographies.

## 2 Methods

The analysis was conducted using data from an online survey (cross-sectional design).

### 2.1 Participants

First-year master’s students of an elite Russian higher education facility (Higher School of Economics - HSE University). These students were all enrolled in a course on contemporary childhood studies in the spring of 2022, which is a minor (second specialization) in master’s programs at HSE University. During the course, all the students (46) were asked to participate in an online survey focusing on their life trajectories. The participants were required to be at least 18 years of age. Out of all the invited students, 65% (30) decided to take part in our research and provided informed consent. The average age of the participants was 24.8 years (SD = 4.4). Overall, 24 of the participants were female, and 6 were male. The study participants represented 15 different master’s programs.

### 2.2 Procedure

The survey items were based on the narrative tradition in psychology (McAdams et al., 1996). Specifically, the survey included two open-ended questions that asked the participants to discuss two life situations in a free format essay of 300–1,000 words. Firstly, the participants were requested to describe the situation in which they made a choice that they considered unfortunate and that had a tremendous impact on their current life. The participants were asked to specify what happened, when, where, who was involved in the situation, and what feelings and thoughts this situation caused then and evoked in the present. Secondly, the participants were requested to discuss another situation in which they made a fortunate choice using the same format.

### 2.3 Data analysis

Qualitative thematic analysis (Braun and Clarke, 2012) was used to reveal the types of choices made by the participants. The data were condensed into categories of choices by three researchers. As a result, we obtained five codes for both unfortunate and fortunate choices, including (1) higher education choice, (2) other education and work-related choices, (3) romantic relationships and friendships, (4) relationships with relatives, (5) and moving to another city (Table 1). All coding was done in Excel.

**Table 1:** Fortunate and unfortunate choice types, *n* = 60.

Type	Unfortunate (*n*)	Fortunate (*n*)
(1) Higher education choices	Studying in college (8), program choice (2)	Studying in college (9)
(2) Other education and work-linked choices	Changing school (1), not changing school (1), not finishing education supplementary to schooling (2), supplementary education choice (1), handing in the wrong assignment at university (1), work choice (2)	Changing job (1), work choice (2), choice of university scientific advisor (2), not quitting higher education (2), supplementary education choice (1)
(3) Romantic and friend relationships	Bullying somebody (1), ending a relationship with a friend (1), not accepting a marriage proposal (1), ending a romantic relationship (1), starting a romantic relationship (4)	Continuing a romantic relationship (1), socializing with peers during higher education (1), participating in a festival instead of spending time with friends (1), starting a romantic relationship (2)
(4) Relationships with relatives	Parental divorce (1)^1^, moving out from parents’ house (1), not saying goodbye to a relative who died later (1)	Returning to parents (1), following father’s advice (1)
(5) Changing residence place	Moving to another city (1)	Traveling (1), moving to another city (5)

In brackets are presented the numbers of participants who mentioned certain choice type.^1^Student considered the parental divorce a choice.

## 3 Results

### 3.1 Unfortunate choice narratives

The most frequently mentioned unfortunate choice type was the higher education choice. Overall, 10 out of the 30 participants reported that either their decision to pursue their bachelor’s or master’s degree was not the most suitable decision for them (Table 1 and Supplementary Table S2). Specifically, some participants expressed that their choice of higher education was unfortunate because they had to move away from their native city, where they had warm and supportive relationships with family, friends, or romantic partners:

*“In 2013, I got into the HSE master’s program through the Olympiad. During that period, I was living with my mother in Altai Krai. At the end of August, I needed to go back to university. The tickets were bought, the suitcase was packed, but I got rid of these tickets because I fell in love. I decided to stay at home. My mother and other relatives did not try to change my mind, and it was difficult to make this decision for a long time.”* (female, 32 years old).

Others claimed that their higher education choice was unfortunate because they did not choose the right program. This happened either because of pressure from relatives or misconceptions regarding the program format.

*“I think that my unfortunate choice was to become a public accountant. When I chose this type of education, I wanted to build a successful career and be an independent woman. Therefore, at 17, I needed to move from my home city to a bigger one. My parents always advised me to be a doctor since they were doctors. I was confident in my choice to be a doctor, but I was sure that it would be impossible to be wealthy in that profession. Now, I think that I should have become a doctor; I frequently think that it is a pity that I am not a doctor.”* (female, 39 years old).

Additionally, romantic and friend relationships (8), other education and work opportunities (8), relationships with relatives (3), and changing their residence place (1) were mentioned as other types of unfortunate choices.

### 3.2 Fortunate choice narratives

The reported fortunate choices also mainly focused on significant decisions related to higher education. Overall, 9 students reported being satisfied with their choice of studying at the university. The students explained that this choice helped them to access new resources, such as social connections and work-related opportunities.

*“In 2018, I decided to go and get a master’s degree in France. However, I did not have money, and it was impossible to get help with that from others. Furthermore, this project seemed outrageous to me, and I was scared. Now, I am very happy that I made such a decision. At that time, I thought that I was going on an adventure. Now, I understand that it was a brilliant decision that made it possible for me to access a lot of developmental paths and helped me find the most interesting field for me. That choice defined my future.”* (female, 24 years old).

*“Following my graduation, I found myself at a loss about my next course of study. I had no idea what I wanted, and “advisers” from all angles were pressuring me, which made things worse. I also felt uncertain and had low self-esteem. I chose to compile a list of universities and a “short-list” of bachelor programs that could probably be applied when completing the budget using my points from the unified state test. Eventually, I managed to find that location and path, for which I am incredibly appreciative. I now see this as the beginning of my capacity to organize knowledge, think critically, and analyze in a way that has evolved with me.”* (male, 24 years old).

Other fortunate choices related to work and education (8), changing residence place (6), changes in relationships with friends or romantic partners (5), and changes in relationships with parents (2).

## 4 Discussion

Mostly, the young people reported failing in their educational choices. The reasons for those failures were moving from their native city, a lack of close relationships in the new place, pressure from relatives in choosing a studying program, and ignorance regarding the program format. Regretting the wrong choice of educational program is common, as young people try different spheres of life, determine their preferences, and develop their life experiences. However, students from countries in which the educational trajectory can be changed do not perceive this choice as fatal (Kucel and Vilalta-Bufí, 2013). In Russia, there are specific expectations regarding the educational backgrounds of specialists based on the profession pursued (Minina and Pavlenko, 2022). As in the example given, becoming a physician takes many years of prior education, and it is hard for middle-aged people to undertake this (Galkin, 2020; Temnitskiy, 2021). Another narrative focused on career choices in design or social sciences, which seem to be much more accessible even to people who are in the period of “established adulthood.” We suppose that, in order to make the education of physicians seem a more financially prosperous path, more money should be spent on financial aid, and stereotypes about the economic aspects of being a physician in Russia should be addressed (Temnitskiy, 2021). However, surprisingly, educational choices were most often evaluated as fortunate. In this study, as in previous research, we found that the majority of students reported their choice of university education as fortunate because it positively affected their career prospects (Hemsley-Brown and Oplatka, 2015).

Overall, the same life events can be marked as fortunate or unfortunate, and this can be explained by personality differences. For example, students in more negative emotional states are more prone to classifying certain events as unfortunate (Rakhshani et al., 2022). Furthermore, the events mentioned were all in line with the findings of studies on major life events; however, unlike in the latest studies, the events mentioned by the students did not include legal, health, and societal issues (Haimson et al., 2021). Furthermore, in comparison to Haimson et al. (2021), starting college and graduation were more likely to be perceived as positive events. Relocation to another city was perceived heterogeneously, and relationships were found to be the most heterogeneously perceived area in the assessment; indeed, they involve a significant amount of uncertainty and dependence on other people.

If we compare our findings with the previous literature on choices, it is clear that in the students’ narratives, structural and cultural factors as the limits or the sources of possibilities were almost not acknowledged. This means that the students did not perceive their choices to be particularly affected by societal norms and expectations. Therefore, according to the students, socioeconomic status, regional well-being, and other elements of the social structure did not significantly affect their decisions. Conversely, interpersonal effects such as parental advice and personal factors, including interests and self-esteem, played important roles in their stories. We assume that the influence of the macro contexts that impede or foster choices are hidden from people (Laughland-Booÿ et al., 2015; Minina and Pavlenko, 2022). For instance, an internal locus of control may lead to self-esteem distortions (Wang and Lv, 2020).

### 4.1 Limitations

Firstly, we cannot clearly distinguish the students’ interpretations of their choices from the influences of educational context, social desirability, and other factors. Context could have caused the participants to remember primarily education-related choices, and social desirability may have facilitated them to hide unpleasant situations and disclose only the ones for which they would not be judged by others. To increase the validity of the results, it would be more reliable to use unsolicited personal data in future studies (Jones, 2000). Secondly, the gender is a factor that influences the perception of life events, but it was not examined in this study, so in the future, it is necessary to evaluate the contribution of this variable to the construction of narratives. For instance, males are more likely to experience stressful life events related to achievements, whereas females are more likely to experience stressful events connected with interpersonal experiences (f.e., caregiving) (Cohen et al., 2019). Finally, the conclusions obtained cannot be extended to the whole population, because only students of one Russian university took part in the study. In future studies, it would be useful to increase the size and heterogeneity of the sample in order to assess the contribution of socio-demographic variables.

### 4.2 Implications

Our findings can be used by university career counseling services. Clients of such services may be advised to write narratives about their successful and unsuccessful choices, as this may help them better reflect on their achievements and failures. In order to make recollections regarding unpleasant life situations less self-centered, self-blaming, and traumatic, career counselors should advise students to think more on the external factors unrelated to themselves that may have caused these failures. In addition, as an example of possible ideas for implementing writing in career counselling, we provide some worthwhile writing practices for university students counselling in the Appendix.

## Data availability statement

The raw data supporting the conclusions of this article will be made available by the authors, without undue reservation.

## Ethics statement

The study does not go against the ethical guidelines set forth by HSE University (https://www.hse.ru/en/org/hse/irb/ethics). The studies were conducted in accordance with the local legislation and institutional requirements. The participants provided their written informed consent to participate in this study.

## Author contributions

All authors listed have made a substantial, direct, and intellectual contribution to the work and approved it for publication.
