# Supplementary material for: Education, relationships, and place: life choices in the narratives of university master students
Source: Front Psychol. 2024 Apr 17;15:1232370. doi: 10.3389/fpsyg.2024.1232370 (PMC11061474; doi:10.3389/fpsyg.2024.1232370)
Supplement: Supplementary file 1 [file Data_Sheet_1.docx]

Supplementary Material

# Tables

The manuscript's authors coded each article independently before getting together to debate and decide on a consensus.

**Table 1**

*Semantic features of essays based on the Harvard IV-4 dictionary, n = 60*

- The analysis was done automatically using SÉANCE (Sentiment Analysis and Cognition Engine). <https://soletlab.asu.edu/seance/>
- The table is too huge to display; however, you can access it using this link: <https://docs.google.com/spreadsheets/d/1VaIi3YYoIU68WnSSnXfTdJPOJEGDlIyNM6by-MRblQc/edit?usp=sharing>
- For interpretations of all coefficients, visit <https://inquirer.sites.fas.harvard.edu/homecat.htm>.

Using the SÉANCE analytical tool, all texts are examined based on the identification of speech markers unique to the text. The table's columns, beginning with "Positiv," each indicate the fraction of words in a given text that fall into a particular category. The number could be between 0 and 1, with a closer value to 1 indicating a higher percentage of the text's words falling into the category. Cell "C2," for example, reveals that 5.9% of the first participant essay—which is devoted to fortunate choice—expresses a positive tone. Accordingly, this essay about fortunate choice at the linguistic level basically lacks the presence of phrases that scholars have linked to a positive tonality of speech (see https://inquirer.sites.fas.harvard.edu/Positiv.html). It is preferable to compare participants and essay categories with one another when examining the numbers in the table. For instance, it is evident that in his account of his fortunate choice, respondent 5 (Cell C10) was more than twice as positive as respondent 1.

**Table 2**

*Coding on events and choice types, n = 60^[[1]](#footnote-1)^*

| Participant ID | Unfortunate choice |  | event | Themes |  |  |  |  | Fortunate choice | event | Themes |
| --- | --- | --- | --- | --- | --- | --- | --- | --- | --- | --- | --- |
| 1 | In 2012, I went to study in the USA with a subsequent desire to continue my studies abroad. At that time, I was 16 years old and at the same time I was finishing an external course at a Russian secondary school (grade 10-11). Having arrived in Russia and passed the Unified State Exam, I could apply for a place in the best universities in the country, however, studying abroad was closer to me. But at the family council, under pressure from the parents, it was decided to also submit documents in Russia and consider this option a priority. It is also worth noting that I began a romantic relationship with a young man who studied in Russia at the Higher School of Economics. At that time, my feelings of affection and nascent love feelings turned out to be stronger than my true desire (to study abroad), as well as the pressure of my parents, on whom I was financially dependent, put an end to it and I entered HSE. Then I thought that this was right for me, Since I have to listen to my parents, my father has authority in my family, I wanted to please him. As for thoughts and feelings now, almost 10 years later, I regret that I didn’t choose myself and my path and was afraid to contradict my parents, and also let my feelings decide (my boyfriend and I lived together for 9 years, got married, and are now getting divorced) That’s why I I realize even more clearly that the choice should not be emotional, but rational, cold and one where YOU yourself are the priority. |  | higher education choice | education, parent-child relationships, romantic relationships, moving, money |  |  |  |  | After graduating from university in 2017 (bachelor's degree), I chose to go deeper into work rather than continue my studies. Despite my parents’ desire to continue my education, I decided that business was closer to me, and I could always continue my education if desired. And then and now I feel a sense of pride in myself, since I feel confident in my field of business. Four years after this decision, when choosing a master’s program, I started from my current knowledge and needs for it, so I consciously chose a program in my sphere and entered with ease. on the budget. What then and now my thoughts are similar - those who do and take risks get it. In my case, I could have easily remained in the care of my parents and studied for a master’s degree for two more years, however, I chose my ambitions and the need for fulfillment. | work choice | education, parent-child relationships, work, money |
| 2 | Quite recently a situation happened to me. In my opinion, getting into such a situation in Russian realities is not difficult. As, perhaps, in other countries (but I did not have to live there on a permanent basis).  By profession I am a modern dance teacher. At the moment I work as self-employed. Modern dance or “free” dance is not the most understandable subject for the average Russian citizen - this is evidenced by my experience of interaction with student actors of Russian universities, with students - choreographers of 1/2 courses, including teachers. Every time I make enormous efforts to involve in my classes not only people who for some reason are interested in movement (everyday body), but also even dancers, those who call themselves such.  In the industry of children's contemporary dance, as well as for adults, and dance in general, if you are not a male dancer or a male teacher, most of everything is decided by acquaintances (“connections”). If they take you somewhere, it’s through an acquaintance; if they invite you somewhere, it always works well only through someone. But then, when you get older and you can no longer and do not want to tolerate a bad attitude towards yourself, you begin to talk about your rights, and my biggest mistake recently was agreeing to a children's theater project without a contract. All agreements were only in words, when we had already begun interaction on the project. 7 days before the end of the project, when most of the work had already been done, they wrote a message to me (they didn’t even call) that they were stopping interaction with me, since I did not comply with the chain of command. , “children are bored” (and I haven’t heard this from children, and in the end, I’m already a young theater director (director) who is working on adult theater projects within five minutes), other teachers find it difficult to work with me (I also hear this from teachers I haven’t heard) etc. Despite the fact that they continue to use my script.  The conflict arose because the project manager scheduled a general meeting at a time that I couldn’t, and I wrote about this quite politely 3 times in the general chat, then when the time was set, I wrote again, starting with the words “ for those who for some reason did not notice my previous messages, I can’t at this time, but only at 20:20, because I’m busy with something else.” To which a message was received in the general chat that I did not respect subordination and that it was not polite, literally: “beneath the dignity of a leader” to respond to such a message. After which they removed me from the conversation, banned me, and wrote me a personal message asking me to pay only for part of the project. I had several thoughts: firstly, I did not expect the people with whom I had worked for several years in the past to do this. Secondly, you always need to conclude an agreement, because otherwise serious problems may arise. I know that if I had entered into an agreement, I would have avoided this situation. Plus, in the end, I spent more time, effort and my money on this project than I was ultimately paid.  I also came to the conclusion that when working with other people, no matter how much you know each other, you should always clearly discuss the conditions and what may not work. It’s probably good that this situation resulted in small financial losses for me, but at that time they seemed significant. Of course, losing 10 thousand rubles is not the same as losing 200 thousand from the theater when you have almost done your job and you were also deceived. There is a difficult feeling that within the Russian community it is not always clear where to look for support in such cases . As if supporting me in such a situation is not the mainstream of the Russian theater community (children, adults - it doesn’t matter). There is a feeling (perhaps deceptive) that we (as Russian people) in principle do not consider it necessary to share “unsuccessful” experiences. And reflection or stories about this can be considered shameful and certainly not universal, at least in the city in which this story took place. I understand how malicious and angry this can sound both in relation to the city and in relation to the people who live in it (because it happens differently). For 13 years I have been building communication with this place in different ways. And when you are not “one of us” or for some reason stopped being “one of us” - you left, began to do projects yourself and set conditions, stopped being absolutely comfortable, then the first reaction to this manifestation is a lively, sometimes uncontrollable aggression on the part of those whom you I already knew once. And, probably, it doesn’t matter what kind of place it is, what matters is relationships with people and their actions.  I don’t blame anyone but myself for agreeing to such conditions, but sometimes situations seem truly hopeless, when thoughts arise that no one is ready to buy you and your work, qualified, honest, expensive work, unique, but in many ways it is happens because I don’t talk about it properly or don’t write exclusively advertising texts, because I can’t yet fully assign such an “advertising identity” to myself.  The takeaway from the story for me is to always find a way to negotiate shared obligations on both sides. I will not agree to terrible conditions, no matter how many people I know with whom I will work. And for the future (for myself), this is to build and control my processes independently, so as not to depend on those who might treat me unfairly. |  | work choice | work, money, relationships with colleagues, interpersonal relationships, community, aging |  |  |  |  | Moving to Moscow was probably a good choice for me; of course, it was associated with a huge amount of stress, but it really gave me many opportunities. He strengthened me in some “points” that were important to me before, but which were not customary to talk about where I used to live and work.  Of course, this choice affected my life, and moving was not the easiest choice in my life - I had to do a lot to leave, a lot of people to “leave”, a lot of people to say goodbye to, if not forever, then for a very long period and if sometimes the separation seemed like a pleasant factor, then sometimes it was unbearable. I was a participant in this situation; in order to go to Moscow and start studying, I had to leave five different jobs, including the theater institute. I was leaving not only a job that I no longer wanted to do, but also a relationship with my parent that still overtook me in Moscow, but it seems to me that I successfully managed to make them neutral. Despite the fact that I am already 25 years old, I have worked through my childhood experiences and “traumas” with psychotherapists for a very long time and with difficulty.  I received a greater degree of internal and external security and independence in connection with my move, although I was not immediately able to appreciate it, but only after time. It seems to me that I began to act more honestly towards people and towards myself, because I truly understood the value of friendship and money. When I was in Novosibirsk, it didn’t seem to me that friendship was important, that is, it was important, but I stopped noticing how many people I know here, and how much they did for me, and I for them. My inner state tells me that I began to notice more “pitfalls”, that my communication skills have increased - it’s true that the demands from society towards me seem to have become many times greater. And I have not yet learned to fully cope with these “requests,” but it seems to me that I am moving in the right direction. | moving to another city | moving, interpersonal relationships, parent-child relationships, work, education, friendship, psychotherapy |
| 3 | I don't regret my choices because they all led me to where I am today. But I would still change one situation.  I went to driving school in 2020, 1.5 months before the coronavirus appeared in Russia. Then, during quarantine, everything was closed, the driving school did not work. And I was in no hurry to return after the restrictions were lifted, there were always things that got in the way - work at the camp, sessions, participation in events and projects, volunteer work... It all got to the point that I took the practical exam only in June 2021. And a couple of months before, in April, the rules were tightened. And now, I haven’t been able to pass my license for a year now. I have taken the practical exam 7 times already. And it just doesn’t work. Something always gets in the way: mistakes, inattention, stress and anxiety... And if I had gone earlier, perhaps I would already have a driver’s license (before, if you passed, for example, a race track, at the next exams you only take the city, and now it’s all over again, both the city and the race track)  In June 2021, I thought that changes in the rules would not entail any bad consequences for me and was not worried. I thought I would manage everything and pass it. Now I understand that I was very mistaken and I regret that I did not take the test earlier. I think this situation should have taught me that there is no need to put off important things for later. But this is too hard a lesson for me. I want to get through it as quickly as possible and close this open gestalt. And my mother told me not to delay... She was right, as always. |  | not complementary education | education, parent-child relationships, work |  |  |  |  | In the summer, before 11th grade, my class teacher sent me information that the Sirius Educational Center in Sochi was recruiting for the Literary Creativity program. I have always loved the Russian language and literature, but I never thought that I wanted to connect my life with these disciplines.  I thought for a long time whether to apply to Sirius or not. Still, the program lasts almost a month, and I’m in 11th grade, the Unified State Exam is ahead, and skipping school is not a good option! Scary and too dangerous. Many said that it was not worth it, but my family was sure that it was necessary to apply.  I decided to submit. I filled out the application, but... it was not saved and was not sent. So I tried 3 times. It worked for the 3rd time! On September 7, I received a letter in the mail saying that I passed the training in October. This October was busy and very productive. We studied all day, and did homework in the evenings and nights. But it was amazing! I returned home, full of strength and energy, inspired and inspired! Since then I realized that I love literature and the Russian language. At the university, I was a member of the press service of the student council, student brigade... Then I began to lead the main VKontakte group of all Student brigades of the *** region. And a year later she became the head of the press service of the Student detachments of the *** region. Could this little girl in 11th grade have thought that her choice would change her life so much and show her what was truly interesting and important to her? Don't know. But I am grateful to myself that I do not miss the opportunity and do as my heart tells me.  Now I am studying for a master's degree in marketing and this is also the result of my choice and my courage in the 11th grade. | supplementary education choice | education, parent-child relationships, teacher-child relationships, interpersonal relationships, work |
| 4 | In mid-2020, I persuaded my family - mom and dad - to move from our hometown (***) to the south, to ***. Since the pandemic began, I had the opportunity to work remotely, and my parents were already retired, the event took about 2 months (selling an apartment, buying a new one, moving).  At that moment I was very happy, because it seemed to me that life was starting from scratch: a new city, new opportunities, new places and people. I thought that moving would automatically solve all my psychological traumas and improve my life. But of course, I was wrong. After a couple of months of living in ***, I felt a terrible longing for my hometown. I missed familiar places, native friends, and the atmosphere of the city. In the new city, I felt alien and unnecessary, events and places became uninteresting to me over time, the euphoria passed. I realized that this decision was made too quickly, that it was necessary to solve my psychological problems not by moving, but by going to the doctor. After six months of living in ***, I decided to return to *** (without my parents).  1.5 years have passed since my return, and I still feel shame and guilt both before myself and before my parents for that act. It’s as if I forced both myself and them to change their lives. But I’m trying to get rid of this feeling of guilt: I began to share my feelings more with my parents, told them about this situation, I felt better. I am also actively working on family relationships with a psychotherapist, and we are also working on the topic of that move. Now I perceive that situation as an experience. If we hadn’t moved then, life would be completely different now: with different jobs, different people around, different values. Perhaps that period (summer-autumn 2020) became a turning point in the awareness of life. That negative emotions, fear, shame are normal. That giving up is normal. In general, I believe that there can be no bad choices in our lives. Because whatever decision a person makes, it will be correct and will ultimately improve his personality. |  | moving to another city | moving, parent-child relationships, psychotherapy, loneliness |  |  |  |  | I can call a job change a successful event in my life. I worked in an IT company in the city of *** as a technical writer, and I decided to leave this professional field: I quit and found a job in the field of editing and copywriting in a *** IT company (I started working remotely).  This happened almost a year ago - in August 2021. I was the main participant in the situation, but since my dismissal was also connected with other people, I can also name my former colleagues who to some extent provoked my dismissal as participants in the situation. At that moment I was working in a promising IT company in a good team under good conditions, and it seemed to me that everything suited me. But in the background it always seemed like something was wrong. I was often tired, often felt depressed and irritable at work. But I suppressed these feelings because I convinced myself that my job was perfect and couldn’t be better.  But at one point I realized that I was gradually burning out: they demand too much from me, I am criticized too often, and most importantly: I do not feel interested in what I am doing. I liked to write creative and media texts, and I wrote texts for developers. I made excuses that it was prestigious, but it did not find a response in my soul.  When I decided to quit, I felt terrible fear: it seemed to me that I would never be able to find something better, I did not have enough skills and experience to respond to vacancies. But still, I overcame my fear, prepared my CV and began looking for a new job. When the final interview at the new company was completed and I was invited, the feeling of fear and anxiety became less. But the thoughts that I was unworthy of this still did not go away (imposter syndrome). When I remember that period now, I understand my emotions perfectly: change is always stressful, and it is absolutely normal to feel it, even from a biological point of view. I don’t blame myself for that anxiety, but I try to use it as experience in future situations. The main insight of that choice is that nothing lasts forever and that you can ALWAYS find something better. This applies to work, study, relationships with parents, partners and friends, tastes in everything, etc. It's normal to change something in life, and change is always a new experience that makes us stronger. Therefore, now, when I think about changing jobs or anything else in life, I no longer experience such stress, I accept it as an experience. | job change | work, relationships with colleagues, interpersonal relationships, job change, burnout |
| 5 | I consider my bad choice to be the choice to move to another school in 5th grade. After studying for 4 years in primary school, I acquired a large circle of friends and acquaintances in my hometown near ***. I was always at the center of the company, a model student. But my parents wanted me to enter a more prestigious gymnasium in ***. Although the choice to send me to a stronger school was theirs, the choice to pass the exams and move or fail and stay where I was mine. I decided not to upset my parents, who diligently prepared me for the exams and convinced me that it was for my good.  As a result, after successfully passing, I entered. As a result, due to the way the class was recruited (most of the applicants were from a pro-gymnasium and studied in the same class), I turned out to be one of the few “loners”, which resulted in further problems - attempts to “establish a foothold” in a new society in which I never felt like I belonged. I wasn't a victim of bullying, but I always felt like I didn't belong. Once adolescence began, I began to have serious academic/behavioral problems and began to become increasingly withdrawn from most of the class.  Back then, I had very conflicting feelings. I didn’t want to leave my hometown, my close circle of friends. On the other hand, I felt a certain “responsibility” that my parents then imposed on me, that I should go to a better school in order to study better, etc. Then, I thought that this would be more correct and better for everyone. Essentially, I made a choice between feelings for my native place and between a certain “voice of reason.” Now my feelings also remain contradictory. On the one hand, I understand that I lost a lot by studying at a new school in grades 5-7 - I sank very much socially, and although I blame this primarily on my parents for neglecting my desires for the sake of my ambitions, I understand that part of the blame lies with me, since the final choice in the form of passing the exam was mine.  On the other hand, in 8th grade I moved to another class and met great kids with whom we are still friends. Life got much better in high school. But was it worth it - in my opinion, no. And some better education at the new school was definitely not worth it. |  | changing school | education, parent-child relationships, relationships with peers, friendship, school change |  |  |  |  | I can consider my choice to go to the undergraduate freshman reunion to be a good choice. Being an introvert, it’s quite difficult for me to force myself to make new acquaintances, but then, in August 2017, I decided with great difficulty that I needed to go to a meeting in Gorky Park at all costs in order to get to know my classmates better. As a result, the meeting went great - I met at least 10 people with whom I subsequently communicated well throughout my studies. Surprisingly, I felt quite comfortable around new people - after a little activity in the park, a small group of us went to a hookah bar, where I tried smoking for the first time. Now I remember those events with a small smile, because... it was almost 5 years ago, and time flew by very quickly. Unfortunately, I never made close friends during my studies, but communication with my classmates in the future was very pleasant. This meeting gave me a big social “boost” in further communication; I consider it definitely a good choice that I made, despite my laziness.  But, if I look back, I understand that I did not learn the right lessons from that meeting - my reserve of social activity quickly ran out, and I did not hang out with my classmates after classes, but simply drove home every time - most likely because of this I I never made any “close” friends at university. I definitely should have developed my social skills better for my own sake and attended these “meetings” more often. | socializing with peers during higher education | education, friendship, relationships with peers |
| 6 | The choice to go to the volleyball section in the 8th grade: after finishing my “career” in football, which did not make me happy, starting at the age of 13, my father, citing the fact that “it is necessary to play at least some kind of sport,” sent me to a sports school Olympic reserve in ***, where, after the first training, I burst into tears from the feeling of my own insignificance, since all the guys had 5-10 years of experience. I felt helpless in choosing what I really wanted to do, as well as loneliness from the fact that “no one understands me.”  As a result, I almost caught up with the guys in terms of skill level thanks to hard training and work ethic, although I gave up volleyball in the 10th grade after playing there for 2 years. Now I am partly grateful to my father for the fact that I learned to persevere and achieve results “out of nothing,” but I would hardly want my own children to go through a similar experience, since I believe that the best decisions are made independently |  | supplementary education choice | education, sport, parent-child relationships, loneliness, relationships with peers |  |  |  |  | After graduating from school, I was at a crossroads in choosing a further vector of education: I didn’t know what I wanted, and “advisers” were pressing from different sides, which affected my condition, there was no confidence, my self-esteem was floating at the bottom. I made the choice to write out a “short list” of directions that I could go to on a budget with my Unified State Exam scores, as well as a list of universities. With this sheet, I turned to independent “experts” from my environment and my family’s environment to listen to their opinion on this matter, recording all the options on a piece of paper. As a result, I entered that place and direction, to which I am incredibly grateful.  Now I perceive this moment as a starting point in the way of thinking that I have developed, in the ability to structure information and think critically. | higher education choice | education, interpersonal relationships |
| 7 | When it happened: An event that happened to me in kindergarten. I was 5-6 years old (I don’t remember exactly), I had no problems with socialization among children and a new team. I communicated with everyone and rarely had conflicts.  The essence of the situation: In our group there was a very fat boy named Seryozha, for whom I, as a child, felt strong sympathy; moreover, my parents explained to me that his obesity was not associated with overeating and the process of losing weight was difficult for such children. Their words stuck deep in my head.  In winter, we often played snowballs when the teachers took us for a walk. During one of these outings, we got so excited about playing “snowball war” that Seryozha himself joined us. We ran and rushed around the site and, in the end, I found myself next to Seryozha, who prepared an impressive snowball-shaped projectile with which he was going to “crush” me. It so happened that he was able to implement his plan and hit me on the head with this snowball. To say that I was in pain would be an exaggeration; I was very offended that such a clumsy boy could throw a snowball so accurately at my head. In a fit of resentment, I called him either “fat” or “fat.” Seryozha instantly changed his face and quickly moved to an empty part of the playground, away from the game. I instantly realized that I had made a big mistake, since Seryozha did not do it out of malice, he was generally one of the kindest children with whom I interacted. Because my offense was mixed with a strong feeling of shame and sympathy, I ran away from all the guys in tears. Inside, I felt like a villain, a scoundrel and a monster who had insulted the harmless Seryozha, who already had problems with socialization (for obvious reasons). I remember how we were able to continue communicating with him and both forgot about this incident, or, in any case, pretended that we had forgotten.  When I was alone, this situation flashed through my head very often and did not give me peace. More time has passed and we have already graduated from kindergarten. It so happened that before entering school we underwent something similar to a medical examination in the same clinic, but on different days. I was lucky enough to be there on the same day as Seryozha. Then I was finally able to gather my strength and apologize to him for my words when we were alone. I will never forget his kindness and sincerity, because to my apology he replied something like: “It’s okay, we’re friends!” and then hugged me. I was so glad that we were able to talk about it and then go around the doctors' offices together, telling each other about how our summer vacation went. Inside, I still thought that I had offended him greatly with my words, but he simply did not show it. As a result, before we parted, I apologized to him again and said that we had fun.  About 17 years have passed since that incident, but this situation remains so vividly in my memory that I am unlikely to forget it. To some extent, I am glad that this happened to me, because for myself personally, I turned out to be not a villain and not the kind of person who can afford to mock and mock physically or mentally weaker people. Despite the fact that at that moment my choice to offend Seryozha with a word was wrong, I never again allowed weaker children to be offended at school. Moreover, a couple of times I even got into fights because more hooligan children offended the fat boy. This situation sharpened, in a good sense of the word, my sense of justice and the desire to help those who are weaker than me. Now I look at this as a stage in my growing up through a stressful situation. While still a child, I was able to independently draw the right conclusions for myself, no one scolded me for this, the teachers did not notice what happened at all. In that situation, I was completely alone, without parents or anyone who could scold me, however, I partially built within myself a value system in which offending those who are weaker than me or those who will not answer me is unacceptable. |  | bullying somebody | education, friendship |  |  |  |  | In the 2nd year of my bachelor's degree, I experienced an internal crisis, at that time I succumbed to atheism, many fundamental questions arose inside, such as: does God exist? Why is there so much evil around? Who do I see myself as? Did I do the right thing by entering the place where I am studying? Will I be able to find myself?  This condition grew every month, it began in the first year. At first it was in the background in stressful situations, then these questions became louder, and my uncertainty became brighter. In the summer, before the start of my second year, I was faced with a situation where the ability to control my feelings and emotions was “taken away” from me. I was completely immersed in this state.  I’m ashamed to admit, but it got to the point that in October of that year, thoughts about laying hands on myself first began to surface. And the worst thing for me was that these were not emotional thoughts to create feelings of pity for myself or to feed my ego with ideas about how everyone would grieve. These were quiet, “calm” thoughts and I stopped wanting to open my eyes in the morning, get out of bed, and so on. Despite my depressing state, I found the strength to talk to my father, who treated my condition with deep empathy and understanding, without accusing me of exaggerating problems and devaluing them. “Son, from what you told me, I cannot give you specific advice on what is best for you to do, your condition causes me great anxiety, but I cannot voice specific actions, perhaps you should start paying more attention to the spiritual component of your life..." He voiced many more aspects that resonated with me, but I would like to focus on what I quoted above.  At that moment I had a choice: follow his instructions about spirituality or treat it like a complete materialist. I chose the first one, started reading more about the Abrahamic religions, because I wanted to approach the choice consciously. I will not dwell on the process of study itself, since it was scrupulous and thorny, due to my internal questions and eternal attempts to find contradictions in the Scriptures. As time passed, I settled on Islam, since I could find all the answers to my questions there, moreover, the contradictions that existed in my head were also resolved. I began to devote more time to spiritual practices, such as the obligatory daily five times prayer, annual fasting, meditation on the meanings of Scripture, and so on. And over time, my condition became better, I regained the meaning of life. The anxiety that always followed me like a shadow hardly bothered me. This choice radically changed my life and attitude towards it. I feel grateful that I was able to find a way out of the crisis, put myself back together and finish what I started. Moreover, admitting my powerlessness in some situations where all the reasons were created added to me confidence and peace of mind that I had never even dreamed of during the crisis.  Another important aspect of this story is that I trusted my father despite my views. Being able to put aside your preconceptions and open your head to what may initially seem uncomfortable is an important quality that I learned to some extent in that situation. Perhaps this story is overly intimate, I would like to apologize in advance, since not everyone will be comfortable and interested in reading about it, but it is well suited for answering about a good choice in my life. If some parts of the story raise questions, then I would like It should be noted that I tried to shorten this story as much as possible, keeping the main points, in fact there were a lot of details that also influenced my choice. | following fathers` advice | education, parent-child relationships, religion, suicidal thoughts |
| 8 | Looking back, I understand that there were no unsuccessful choices.  With all the pros and cons. Most of my life is studying, and each of the choices of school/university/specialty still gives you something, despite some things that you currently evaluate like something that could have been better But I can’t call it unsuccessful Because Everything should be accepted with gratitude There are only your actions, not choices that you regret In which you would like to change something if you turn back time For example, if about your memories, then I can tell you Late evening A group of friends after a long evening of games finally disperses These are friends who are more like family, brothers and sisters, because you have been with them since childhood And you have known them for twenty years You  are so used to the fact that you see each other from time to time that you think , that this is another evening where you relaxed together and then meet again. Therefore, when you separate, you become too lazy to go up to just your “sisters,” Because you’re too lazy And, moreover, you’ll see each other later anyway, So you, tired and sleepy, sit down in car and go home, Without saying a complete “bye” And then you no longer have a chance to say goodbye to one of the “sisters” Because you go to different cities to continue working/studying And one day one of your “sisters” gets into a car accident Looking back, you want to turn around, come up and hug Or just touch your shoulder, your hands And say “bye” Now there is regret inside you But, unfortunately, it won’t change anything And you just take it as a lesson, you start saying goodbye to people in a normal, different way evaluate meetings both with loved ones and with just good people. Perhaps all this can also be considered as a bad choice  . The choice to say goodbye - not to say goodbye is not that of a trajectory considered in the usual sense; study, goals, work, and so on; a trajectory of understanding life, death and oneself |  | not saying goodbye to the relative, who died later | friendship, death |  |  |  |  | My successful choice was influenced by my sister and brother, as well as simply fate.  It consisted of several mini-elections. The first one was random, summer 2017. Arriving at the Faculty of Philology in another city to submit documents, tired, hungry, and also just wanting to get over it all as quickly as possible. Look. on the list of proposed educational programs within the framework of linguistics And there are 23 of them You ask the girl working in the document acceptance committee to tell approximately what knowledge we will receive in each of the programs The girl calmly begins to explain On the description of the third EP, the brain refuses to listen further As a result, you randomly poke the programs in a different order You put forward OP with beautiful names September 2017 Inspired, you study for the first days as if with wings on your back  and then you receive a call and they tell you that, it turns out, for these two weeks you studied in the wrong program where you are currently attending classes. It turns out that you are enrolled in another one official documents (you yourself randomly poked and didn’t remember where you ended up signing up, so you got confused) And at the EP to which you are enrolled, there is a choice of study - Japanese or Arabic, now you have an evening to think, either you stay where you are studying or transfer to another program, and again you will have to get used to the evening was full of emotions of not understanding what to do. In the end, you resign yourself to your fate and call your elders, That is, the second mini-choice is to call close people who know you well. Sister and brother, of course, told you to go, because there But Japanese! You feel some confusion, you worry (after all, you have to meet people there again) But you still switch In the end, I ended up in another program Got the opportunity to study Japanese Meeting the Japanese And in general, immersion in Asia and now the master’s degree is connected with the East  , looking back, you think How good it is that it’s like this turned out Errors and inconsistencies also lead to something Every choice is a set of factors that lead to another set That's why all this is so important | moving to another city | moving, intersiblings relationships, education, friendship |
| 9 | In 2013, based on the Olympiad, I entered the master’s program at HSE. At that time, I lived with my mother in the Altai Territory. At the end of August I was supposed to leave, tickets were bought, my suitcase was packed, but within a day I handed over these tickets because I fell in love. I decided to stay. My mother and other relatives did not put pressure on me; for a very long time I myself could not make a decision. However, a week later I still went to study and was very happy about it. In general, I believe that there is no wrong choice; in the long run, I always come to the conclusion that I am doing the right thing. |  | higher education choice | education, parent-child relationships, romantic relationships |  |  |  |  | I would give an example of the same event that led to two choices. Finding myself in the first one, when I gave up studying, after a couple of days I realized what awaited me in a small town, or rather, nothing, I was overcome by such melancholy and horror that I immediately changed my decision, even though I was leaving home It’s difficult, it was also difficult to break up with my boyfriend, to settle down in ***, to study and work. But in the end it gave me a lot, a lot. | moving to another city | education, moving, work, parent-child relationships, romantic relationships |
| 10 | In my second year of bachelor's degree (I was 19 years old), I decided to go work as a counselor at a children's camp. I had no idea what kind of work it would be; I thought that working as a counselor would not be difficult. But I ended up in a pile of inadequate “colleagues” who came to the camp to have fun, and not to look after the children. I came across extremely clueless partners, our teacher sat in her room all the time and hardly worked, the second counselor spent all her days drinking with other counselors and teachers. I looked after the children alone, barely slept for three weeks and carried everything alone. But the most stressful thing was the fact that it was impossible to lock the door to our room, so drunken male counselors kept breaking into our room at night. They didn’t cause any physical damage, but yes, they did moral damage. I was very afraid of them, but nothing could be done about it. The camp director told me: “everything that was in the camp stays in the camp, this is how the guys show sympathy.” So I lived all this time, afraid to fall asleep (and there was almost no time, planning meetings ended late, then I drew posters, and then after 3-4 hours I had to get up and gather the children). Then I was constantly scared and bad, I cried, I wanted to go home, but I couldn’t leave, my practice at the university depended on it. A lot of stress had a detrimental effect on my health, I became very ill and was treated by doctors for two years. Now I think that I shouldn’t have taken everything so seriously, nothing terrible happened. But it was still not worth going. |  | work choice | work, relationships with colleagues, interpersonal relationships, psychotherapy, health, loneliness |  |  |  |  | A year ago I decided to enroll in a master's program in ***. At first it seemed to me that this was impossible, I had never been distinguished by outstanding abilities, so I thought that I couldn’t, but I wanted to try myself. My friend, with whom we studied, decided to enter HSE and I simply followed her. Miraculously, I entered the budget, it was truly a miracle, because I graduated from a very average university, moreover, I graduated from its branch, which is why the burden of responsibility weighed doubly on me: I was afraid of not being able to cope with such an opportunity. Here a new question arose - relocation. I had to pull myself together, burn all the bridges in the old place and go to a new life. At first it was very difficult: my body resisted a lot of stress (I was sick a lot), I couldn’t find a common language with my neighbor and I felt terribly lonely. I even thought about leaving everything and moving back. But after 4 months, I suddenly got a second wind: everything improved in all areas, it became easier to live and create. And I decided that I needed to stay and continue to fight. Now I compare my life there and in *** and understand that my life has changed qualitatively and has become better. I no longer regret my decision, I think I did the right thing. I'm getting an excellent education and have a great job. But sometimes, in moments of extreme fatigue, thoughts of going back still overtake me. | not quitting higher education | education, moving, work, friendship, health, relationships with peers, loneliness |
| 11 | 1) unreasonable choice of the direction of study  2) July 20173) parents, young man 4) *** 5) a feeling of uncertainty, lack of understanding of what to do next and how to build your life 6) the inability to change your choice causes a feeling of constant regret (“What if I were you smarter then?)7) thoughts on how to get through this stage faster 8) trying to justify my choice, blaming the indifference of my parents in determining my professional path |  | higher education choice | education, parent-child relationships, romantic relationships |  |  |  |  | 1) moving to ***  2) August 2017 3) me and a young man 4) *** 5) a feeling of freedom, a sense of perspective 6) a feeling of losing oneself 7) thoughts of returning to my hometown 8) no matter what is done, everything is for the better | moving to another city | moving out, romantic relationships |
| 12 | For several years I have been preparing to enter the theater institute. My mother did not support this idea, but did not prohibit it either. My desire to enroll did not disappear, almost every evening I disappeared at rehearsals for the graduation performance, and my studies at school kept getting worse, which frightened my mother. Therefore, we “agreed” that I could attend classes at the theater judge and prepare for the graduation performance if I simultaneously prepared for the Unified State Exam. From the 10th grade, I thoroughly prepared for the 7th exams, since for the theater institute I needed 2 subjects, I prepared for them on my own, and to prepare the rest I studied with tutors, for whom my mother paid. By the end of the 11th grade, I was overwhelmed with final exams, the Unified State Exam, a graduation performance and admission to universities. According to the proverb “if you chase two hares, you won’t catch either,” the quality of each of the undertakings suffered. We went to *** together to submit documents. The first was a theater university, and we only made it to the 2nd wave of admission, where we recruited groups. I didn't pass. Out of the entire wave (about 80 people), only one boy was caught, so my chances were initially extremely small. In the end, we chose a university and I entered comparative studies at the Faculty of History and Philology at the Russian State University for the Humanities. After 5 years, I can't say that I regret this choice. This road led me here, and I would be completely different if I had chosen a different path. But sometimes I’m sad that, under the yoke of parental approval, fear of risk, and lack of self-confidence, I missed the opportunity to try to enter a theater university again. |  | higher education choice | education, parent-child relationships |  |  |  |  | It so happened that today is my boyfriend and I’s anniversary (2 years since we’ve been together). On this day two years ago, I consciously changed my life. I had practically no friends left in my hometown, since at that time I had already been studying in *** for three years and my whole life and social circle was connected with this city. I only came to my parents. During quarantine, I came home as usual. I was bored, and suddenly an old acquaintance from school wrote to me. We met once, then twice, a couple more times. At some point, as usually happens, I realized that I was starting to like him. A little later I noticed that it was mutual. I didn’t feel joy, rather fear, because I knew for sure that he wouldn’t leave Penza, and I didn’t want to stay at all. Therefore, before the next meeting, I thought through our conversation. The idea that I was going to convey (I even rehearsed it!) was that I like him, but I don’t want to meet “just like that,” and it’s unlikely that anything serious will work out for us. Keeping the words I had learned at the ready, I went for a walk. And so, in one of the romantic moments, looking at the stars, I said that we couldn’t be together. I remember that he was confused then and asked: “How can you know this?” The question was so simple that my entire rehearsed speech immediately seemed stupid to me. I suddenly felt that right now I was the one changing my life. As I decide in the next couple of seconds, so it will be. At that moment I made a decision that for several days left me in doubt, seemed irrational, infantile, but today I am incredibly glad that I made this choice. And now my plans for the future have become much brighter than when it seemed to me that everything was thought out to the smallest detail, and there was no place for a guy from my hometown in my life. | starting romantic relationship | moving out, romantic relationships |
| 13 | When the time comes to think about your “unsuccessful” choice, you have to delve a little into yourself and understand at what period in your life something went wrong. Despite the fact that I am a supporter of the fact that everything in this life happens for a reason and even from a bad choice you can always learn a lesson for the future, I will still formulate an example of my bad choice.  I had to delve into myself for a long time to remember my bad choice, so I’ll write the first thing that came to mind. Probably, my worst choice was associated with early growing up, or rather early moving away from my parents. From a very early age, I always wanted to become older, which is probably why I spent a lot of time with guys older than me. My desire led me to the fact that from the early age of 17 I began to live alone (I think this is early) and, probably, because of this, I missed some moments of childhood. Once, when I was 15 years old, one of my friends said: “Why are you in such a hurry to live?” Previously, I didn’t attach any importance to this, but I remembered this question a few years later and wondered, really, why? Generally speaking, there is nothing special about this, but for myself I understand that I missed a certain stage of growing up, formation and “happy” teenage moments, while wanting to grow up as quickly as possible. Now I’m 23 and all my close people say that I’m an adult child who doesn’t want to grow up. |  | moving out from parents | parent-child relationships, friendship, moving, interpersonal relationships |  |  |  |  | Probably my most “successful” choice is partly related to the “unsuccessful” one. When I was 17 years old, I moved to another country to study, completely alone. It was 6 years ago. At first, it seemed like an unspeakable happiness to me: nature, new acquaintances, even a tiny dorm room seemed incredibly beautiful to me - after all, it was just mine. After a while, the rose-colored glasses began to fall off and the understanding came that everything was not as rosy as I thought. The new acquaintances turned out to be not so pleasant; there were the first betrayals and traumas. The nature around was replaced by an eternal fog, and the tiny dorm room seemed unbearably cramped and stuffy. At that moment, I realized for the first time that I terribly missed my family, friends, my childhood bed that I could barely fit into, my mother’s soup, and even music in my native language. Time passed and I began to understand that the “new life” was gradually breaking me and I was no longer the same “Masha” that I was before. Then I thought, what will happen next, another year or two of study? And then, for myself personally, I realized that I couldn’t stay there any longer, that I wanted to go home and it didn’t matter to me what the consequences of this decision would be. I remember very well a call to my dad, which was very short and monosyllabic: “Hello, when I fly home for the holidays, I won’t fly away anymore and will stay at home, I don’t like it here, I’m sorry.” I can’t say that this decision was difficult for me, because I really wanted it; rather, I had to deal with the consequences of this decision for a long time. I remember how I packed all my things and within 3 days I was already sitting on my suitcases. Returning home, my little children's room, my mother's soup and my father were waiting for me, who had not spoken to me for another month, because he did not understand how it was possible to leave one of the best universities in the world. Returning home, I had to reassemble my past life using puzzles, although I had only been gone for six months. But I understood perfectly well that this was my choice and I really wanted it. Quite a number of years have passed, and now I understand perfectly well that perhaps it was my best choice to leave there and return home. Having returned and overcome all the problems, everything worked out for me in the best way: I re-entered a good university, I realized that my friends here are real. And I am absolutely grateful to myself that at that moment I made such a GOOD choice. | returning to parents | moving out, education, parent-child relationships, living abroad, relationships with peers |
| 14 | In the winter of 2021, I met a young man who wanted a serious relationship that would lead to starting a family. At that time, I still lived in my hometown of *** , but even then I understood that I wanted to enroll in a master’s program at the Higher School of Economics and move to ***. I understood his intentions and did not hide mine, I told him about my views and that I did not want to start a family in the near future and settle in *** . To which they responded approvingly, and he convinced me that it would be easier together in a new city, he himself doesn’t mind moving, and there is nothing constraining in the relationship and only joy. After talking with him for a couple more months, I believed every word and fell in love with him. I express my love with help and support, and that’s why I invited him to move into my apartment, gave him the use of my car and helped him find a job with good conditions. For some time, our life was like a fairy tale, until summer came... I started collecting documents for admission and taking entrance exams, I was never afraid of difficulties and had long ago agreed with the idea that I would have to leave all material wealth or transfer them into assets and start all over again in ***, and he was not going to part with comfort so easily. At first, he increasingly mentioned that my hopes and desires were not justified and that I was doing everything wrong, then he began to say that nothing would work out anyway, then he began to put pressure on pity, saying that family and friends would remain here and I would they are far away. All these chants were accompanied by constant alcoholic intoxication and frequent shouting. He tried to turn my parents against my decision, but after failure, while heavily intoxicated, he left in an unknown direction at night. I felt broken and empty, tears flowed naturally. In the morning he called, saying that he had walked home, but instead of an apology, new accusations began to fall about my selfishness and the fact that I did not take his interests into account. We discussed everything and decided to try it anyway, but not to move into the void, but to find an apartment, a job and prepare everything in advance. Of course, I also dealt with these issues; requests for promised support, persuasion and an open cry that it was hard for me were not heard. I still managed to find several vacancies and at the end of August I went for interviews, and while I was away, unknown people came to my apartment with alcohol, which I found out through the video intercom application on my phone. I kicked him out of the apartment, changed the locks, gave the car to my mother and moved to ***. This story made me very disappointed in men. Thinking about it today still irritates me. I’m very sorry for the wasted nerves and energy, maybe if I hadn’t agreed to enter into this relationship then, I would treat men differently today. |  | starting romantic relationship | romantic relationship, education, work, loneliness, moving |  |  |  |  | In 2021, I decided to enter HSE and move to *** from *** . Not without difficulty, but still managed to achieve what I wanted, move, and also get an interesting and promising job at the Federal Tourism Agency. I was delighted when I saw the enrollment list, and after they called me back from work and invited me to come out on a working day, happiness knew no bounds! I like the richness and rhythm of this city; I feel like I’m in my own territory every day. During the time spent here, I made several good friends, on weekends I explore more and more the capital of our homeland and travel around the country. Of course, there are difficulties, but I cope with them playfully and work tirelessly on myself! I see the potential and prospects for my further development and this makes me happy, I really like learning new things every day and increasing my potential. I believe that enrolling and moving significantly sped up my getting on my feet. | higher education choice | moving out, changing city, education, work, travelling |
| 15 | An unfortunate choice that significantly affected my life was my decision to completely withdraw into family life after finishing my undergraduate degree. This happened 3 years ago. It is difficult to name the participants in this situation, since it was my conscious decision. However, this was indirectly influenced by my religious views and the desire to enter into a life together with my then future husband. The final choice not to continue my development in the scientific field was made after defending my thesis. Having protected it at 9, there was a conviction inside that I had done everything I had to do in this area and now I would go a different way. I was returning home after defending my thesis with a young man, and I clearly remember uttering the phrase: “That’s it, I’m so tired over these 4 years that I’ll never return to HSE again.” Then there was a feeling of joy from the studies left behind and anticipation of the future. Regarding my studies, I was convinced that I had done everything to the maximum, I was happy with the successes that I left behind. Ahead I felt a wonderful, measured family life in which I would be in harmony with myself and my husband, I would do whatever I wanted, and I would no longer be in an eternal state of stress and pursuit. Now, analyzing my choice, I feel regret. I know that I could achieve much more if I continued to study and develop myself. But at the same time, I remember my psychological and emotional state and understand that it would not be so easy. There was no strength at all. The next 2 years without training also gave me a lot. I tried myself in business, tried to immerse myself in my family and everyday life, and did things that interested me. But most importantly, I learned to listen to myself and understand what I really want. Sometimes it is worth taking a step back to understand your thoughts and desires. For me, apparently, it is important to find a balance between family, study and scientific activity. And the main thing is that I receive support in this from my loved ones. It’s not scary to make mistakes if you find the strength to correct these mistakes and follow the path that you feel in your heart. |  | starting romantic relationship | romantic relationship, education, work, loneliness, moving |  |  |  |  | I’m not sure if this can be called a choice, but I consider it a great success to choose my supervisor. In the second year, my classmate and I were looking for a teacher who would teach our group coursework. We were sent an Excel file in which the teachers indicated information about themselves and their scientific interests. We were just sitting at a lecture on statistics. We studied the file for a long time and agreed that we needed to write to someone whose topic was close to our interests. The teacher agreed to take us and that moment turned out to be very significant for my future life. I didn’t fully understand this then. I perceived her as an ordinary good scientific supervisor who guides, advises, and supports.  By the end of 4 years, I had more experience interacting with her and had vivid feelings of gratitude and regret that it was all over. After defending her thesis, she came up to me and said: “I will be glad to see you in the master’s program, you will succeed.” It was not taken seriously at the time, but the memory of these words remained for a long time. And in those moments when I couldn’t find myself, didn’t understand what I really wanted, I often scrolled through them in my head. I think that her faith in me gave me a lot, and most importantly, it allowed me to believe in myself, in my strengths and do things that I would not even think about.  My feelings today about the choice that I made, albeit not fully consciously, are very reverent, filled with a feeling of gratitude and joy. In my opinion, finding such a person is very rare. And I am very glad that my choice turned out to be this way. Once again I am convinced that a friendly, comfortable, supportive environment can endlessly fuel a person to grow and move forward. And it's wonderful that this happened to me. | scientific advisor choice | education, teacher-student relationships, relationships with peers |
| 16 | When I went to university, I became friends with a girl who became the sister I never had. Over the course of 4 years, our relationship became stronger: we shared secrets, traveled, laughed a lot, cried together, walked until the morning, spent sleepless nights together over deadlines - in general, we lived the best student life. About a year ago, when we were finishing our bachelor's degree at the National Research University Higher School of Economics in ***, small quarrels began to occur in our friendship; they had happened before, but they were always quickly resolved. Immediately, whether it was the new environment that appeared to us, or whether we grew up and had already exhausted these relationships, I don’t know, but everything began to change dramatically. We had a telephone conversation, with awkward pauses that had never happened in 4 years, she asked me: “What’s going on? I feel like something is wrong. Share with me, please...” And I don’t know why, but I said something like “everything is fine, just a lot of deadlines, my head is full of things.” After these words we communicated, but it could not be called friendship. Both she and I seemed to be making attempts to understand the current situation, but apparently not enough. I felt lonely, stressed, and it was very strange for me that I could not or was even afraid to share my thoughts and feelings about this situation with her. And then I felt jealousy (friendly) when she began to communicate with the girl who appeared in our environment. And because of our “quarrel” with her, the general circle of friends seemed to take her “side”. I saw photos of them together on social networks, and I was removed from the conversation where we communicated for no reason. All the time I thought what I had done that they didn’t communicate with me, it seemed like I hadn’t done anything, but why then does what happens? I don’t think I’ve ever felt as lonely as I did then. Almost a year later, I understand that if I had stepped over myself and answered that simple question of hers “what’s going on?”, I would still have my best friend, I would not have lost the circle of friends that I had developed over the course of 4 years . But despite these thoughts, I let go of the situation and began to perceive it as an experience. And even if it sounds banal, I am somehow grateful for this opportunity. Now I feel that perhaps he helped me look at myself and my surroundings differently. Understand what I want to give to people in friendship and what to receive in return. |  | quitting relationship with friend | friendship, interpersonal relationships, education, relationships with peers, loneliness |  |  |  |  | I think that entering HSE in 2017 can be called such an event. It's a little strange to realize this now, but 4 years of university life gave me so much. Remembering these events now, I don’t even believe that this happened to me. A friend told me about HSE in the 11th grade and after going to the pre-school education I realized that this is exactly the place where I want to study. Mainly because of the program, which was and is now, as far as I know, in Russia only at HSE. I also liked the atmosphere then, but it was not the main factor. When the time came for admission, I submitted documents only to the HSE. And here I want to note that I am immensely grateful to my parents for giving me the opportunity to choose my own university, direction, etc. They supported me even when I entered commerce. I remember my dad said something like: “I’m ready to pay this money, as long as you like it and study is useful.” I think if it weren’t for their support then, my life would have turned out radically differently now. I was very scared then, like any schoolchild upon admission, but at the same time I was incredibly interested in what awaited me there. And the first thing that struck me was the people. Guys from all over the country, with their own tastes, ideas, views. It was incredible then to feel part of this amazing world. But at the same time, it’s a little wild, because I’m not used to the fact that 70% of the people in the room with me have different bright hair colors, and the person sitting next to me is transgender. For me at 17 years old, this was very, very unusual. And then the hostel. At first I didn’t understand what and how. I felt a little lost and worried. I went home every weekend because I still felt awkward and out of place. Everything started to turn around, probably in the 2nd year. A huge number of new people, new events, the first kiss, etc. began to appear in my life. I was already more comfortable being in this new environment, but thoughts still appeared that I could do something wrong, say something wrong. After so much time, I remember with warmth everything that happened, even some embarrassments seem not as terrible as they were then. I came into that new world as a completely small, naive girl, and came out as a grown-up girl who can defend her interests and personal boundaries, who is not afraid to seem stupid if she doesn’t know what is being discussed in the conversation, and without hesitation can ask a clarifying question. . And it seems like everyone has gone through something similar, but it seems like your story is brighter. I am incredibly glad that back in 2017 I chose the right path to my future. | higher education choice | education, parent-child relationships, friendship, romantic relationships, relationships with peers |
| 17 | I think the only case that could somehow negatively affect my life is the divorce of my parents.  But even the process leading to divorce, I think, also affected me. The first is memory - I don’t remember childhood and generally remember what happens rather with feelings and pictures. Secondly, I have developed a behavioral mechanism of constant playfulness and constant denial of the situation. Joking about anything that may not be a joke or a bad joke. Joking out of the understanding that nothing lasts forever and cannot be taken seriously. As if, instead of understanding why something happens in the form in which it happens. And it is even possible to see in this not a reason for laughter, but something poetic hidden at first glance.   Otherwise, such situations have not happened to me. |  | parental divorce | parent-child relationships, friendship, moving, interpersonal relationships |  |  |  |  | A good choice is to live out of love.  First fall in love with something and then bind it with responsibility.   This is what happened to me when I fell in love with school. I fell in love with sports, philosophy, my wife, children, profession, friends.   The best things happened to me when the situation worked out so that I could leave something I was passionate about and gain something new.   Finally, it’s worth highlighting the family. But it was not my choice, and I simply could not live without the person who eventually became my wife. And now I can’t. And now I can’t live without children. When we are all together, everything is not perfect, but when we are apart, everything is devastated.   So yes, family, and everything else is bonuses. | starting romantic relationship | romantic relationship, friendship, having chidren, work, hobbies, education |
| 18 | One of the unsuccessful choices that significantly influenced my life was the choice of a legal specialty instead of a creative one. After finishing 10th grade, I chose to take subjects at the Unified State Examination that would allow me to enter the Faculty of Law and actively prepared for them throughout 11th grade. As passive participants in this situation, one can single out my parents, who, on the whole, did not participate in my choice in any way (they did not pressure, did not advise), but were glad when this choice fell on the legal profession. Then I felt confident, I believed that in life you need to do what can be useful. But now I feel only slight disappointment, because, having received a legal education, I understand that most likely I will not work in my specialty. Since childhood, I have been involved in creativity - I graduated with honors from the choreographic department of an art school, and during my undergraduate studies I completed painting courses, but in order to immerse myself in this field and fully realize myself in it, knowledge is needed, but time has already been lost and retraining for another I no longer have the strength or desire to specialize. Probably the best solution would have been to leave after the first year of the bachelor’s degree and re-enroll in another specialty, but I studied at a “scarf” at a big discount, and the subjects I took were not at all those required for creative fields. I didn’t want to disappoint my parents and lose a whole year. When I made this choice, I thought that I would like the field of law and there would definitely be something for me, but after studying and completing an internship in the state. organs, I realized that all my ideas about this type of activity are very idealized, in life everything is different and I don’t see myself in such a place. However, I think that the education I received will not be in vain - after all, the legal profession is really useful, you never know how life will turn out, so now I rather do not regret it. Through trial and error, I found a little in law that was of interest to me in one way or another and continued to study it in graduate school, since it allows me to realize my creative impulses, at least to a small extent. To summarize, I would say that, having greatly regretted and been disappointed at the beginning of the journey, now I am trying to look for positive aspects in this. Having previously thought that I was wasting years on something I didn’t like, now I believe that one way or another it was a useful experience that will still help me realize myself in the future, although not in the way expected. I will never know how my life would have turned out now if I had entered a creative field, but perhaps the future will show me that the choice that I now consider wrong was the best. |  | higher education choice | education, parent-child relationships, work |  |  |  |  | I believe that one of the best choices of my life was made during my undergraduate studies, and that was the choice of my thesis advisor. The first year turned out to be very difficult for me - adaptation, living with strangers, making new acquaintances, high workload - all these circumstances put a lot of pressure on me. I was very confused, I couldn’t get into the right rhythm of life. Choosing a topic for my coursework also became a challenge because I didn’t know exactly what area I wanted to delve into. I felt insecure and depressed. Moreover, in the first year, one of the subjects in my group was taught by a girl who had entered graduate school. Compared to many teachers, she stood out because she helped in every possible way with advice; you could always turn to her for help even on “stupid” educational questions, for example, what exactly should be in an essay or how it differs from an essay. I chose her as my supervisor, although I didn’t particularly like the subject, but then I gained some support and felt much more confident. While working with her, I stopped feeling like a “dunno,” especially compared to the guys who competed in the Olympics. And it was she who showed me that science doesn’t have to be boring. Although the work on my first coursework in my life was difficult, it determined my scientific interest for the next three years - although now, in my master’s degree, I am working within the framework of this topic. This girl became my permanent supervisor until the end of my undergraduate studies. She also invited me to work with her as a teaching assistant, which allowed me to become more familiar with the work of a teacher and look at the educational process from a different perspective. Together with my supervisor, I also took part in three projects, and I still use the knowledge and skills gained while working on them. If in the first year of my studies I thought that I would only waste time doing an unloved activity, then she showed me that even in it you can find something that will be close to you. It is largely thanks to her that I am now studying for a master’s degree with a feeling of great gratitude. Unfortunately, for certain reasons she had to leave teaching for a while, but I keep in touch with her even now, although another teacher became my supervisor. It may seem strange, but it was this person, without even knowing it, who helped me cope with the feeling of enormous uncertainty and fear of the future. And although throughout my studies I did not like my specialty, now I think that if I had not entered it, I would never have met such an excellent teacher who opened up new perspectives and opportunities for me. | scientific advisor choice | education, teacher-student relationships, relationships with peers |
| 19 | Changing eating habits and attitudes toward food after experiencing peer pressure.   I was 16 years old, and for the first time my sister and I went to a children’s camp at sea. Our environment included previously unknown guys from completely different walks of life. Like kids from an orphanage, wealthy families, guys with average income.   Girls my age often talked about being overweight (with a height of 180, I then weighed 68), constantly teasing and persuading the boys not to communicate with me. At evening events, no one came up to dance with me, which made me very sad. I didn’t understand why none of the boys wanted to communicate with me, and how my weight could affect this, if in my understanding I was not chubby. I was very sad and felt injustice. After this incident, when I arrived home, I began to lose weight urgently, which greatly affected my health. I gave up meat and as a result, due to my growth, the bones did not form normally, which led to further surgery on the spine.   Now I have a negative attitude towards this situation; I consider people who bully others for any external differences to be abusers and do not allow such people to have any contact with me. |  | starting romantic relationship | relationships with peers, bullying, heat |  |  |  |  | I took part in a dance festival when I was 15 years old. there was an important rehearsal that I had the choice of going to or staying and hanging out with friends whom I hadn’t seen for several months because they had moved to another country.  I decided to go to the rehearsal, which later helped me take a prize and win a subscription to a very cool dance studio. Then I thought that I had made a stupid decision, because I really wanted to hang out with my friends and spend more time with them. I was ashamed in front of them that I had to leave so early. Now I’m very glad that I chose the rehearsal, it brought me a lot of experience and further development. | participation in festival instead of spending time with friends | hobbies, relationships with peers, competition |
| 20 | At the age of about 9 years old, I decided to enroll in a music school. Before that, I studied with a tutor at home for quite a long time, and by the time I enrolled I already had certain basic skills in playing the piano. After successfully passing the entrance exam, I had the opportunity to skip the preparatory year and go straight to the first grade of a music school.  After a few months of classes, it became clear that my relationship with the teacher in my specialty was not working out. Every year it became more and more difficult to attend them, each of them was accompanied by great excitement, stress and even fear. There were many children around who felt the same emotions, so at that moment it seemed to me that this was normal, because many of the children are in this position, so it’s worth being patient. But there were other examples when communication with the teacher and the classes themselves gave my peers pleasure and pleasant emotions - I kindly envied them.  After finishing sixth grade, I went on a business trip with my parents for several years. At that time, it was decided to take assignments in all subjects from all teachers, work on them and, returning in the summer, pass all exams and graduate from music school. Throughout the school year, I was very worried about going back to school. At that time, I was already about 15 years old, but the childish panic fear of classes and the teacher persisted. In the end, my parents and I made a joint decision to leave studying at a music school, and with it all the fears and worries in this regard. After that, I felt unprecedented relief and finally let go of the situation. Now I understand that if I returned and finally put a final point in my training, I would have the opportunity, if not to overcome my fear, then at least to prove to everyone, and most importantly, to myself, that I am able to make a strong-willed effort on myself and finish such a difficult path, despite all the troubles and difficulties that I had to face.  If I had to make a choice now, I would certainly do things differently. Of course, such moments build character and train the child’s will (an adult understands this, but not a child, when he is in the epicenter of a situation), although from a psychological point of view this can be very difficult. |  | not finishing education supplementary to school | education, parent-child relationships, teacher-student relationships |  |  |  |  | I consider entering the *** State Law University named after O.E. one of the most successful choices of my life. Kutafina. In 2017, after graduating from school, my main goal (and even dream) was to enter the law faculty on a budget. The process of receiving the Unified State Exam results, submitting documents and waiting for the final lists was very exciting. When I saw my name on the lists, I was absolutely happy, and my parents had the same feelings. At that time, I believed that a successful future awaited me, but I hardly thought about any more substantive issues, learning difficulties, or passing state exams. There was only euphoria from the fact that my main dream had come true.  Studying throughout all four years was very difficult, associated with a huge amount of stress. Family support and the realization that at the same time there were a huge number of the same students studying with me, who were in the same conditions as me, helped a lot.  Several times I really wanted to give up, when the motivation to work completely disappeared, and the efforts made seemed disproportionate to the results obtained. At such moments, it was very difficult to concentrate on understanding that this is one of the most important life lessons, I’m not afraid of this word, a “school of life”, which was very useful to go through. I am very glad that at such moments I was able to maintain clarity of mind, compare all the pros and cons and complete your studies. Having received my diploma, I certainly felt proud and thought that now I could confidently move on, because with such experience I would probably not be lost. I have friends who left university once, or even several times. At such moments I think, what did studying at the university give me? Of course, graduating from a prestigious university does not provide us with a 100% guarantee of finding a well-paid job, nor does it give us any special status, the presence of which would characterize us as an absolutely successful person.  However, I can safely say that over these four years I have learned to make independent decisions, take FULL responsibility for my actions, communicate and negotiate with people and, most importantly, not give up. I am still proud that I was able to go through this stage of life with dignity - it gives me the strength to continue to learn and improve. | higher education choice | education, work, health, interpersonal relationships, relationships with peers |
| 21 | Since sixth grade I wanted to change schools. The one where I studied did not provide much knowledge other than the French language, and, most importantly, bullying flourished there. But my mother, convinced of my abilities in foreign languages, did not allow me to enter other schools where my friends studied. As a result, I entered the school that I wanted to go to and which I still love to this day, only after the ninth grade, in 2012. The bad choice was to listen to my mother and put my entire high school in French, no matter what. The main participants, respectively, were my mother and me. All this happened in ***. From sixth to ninth grade, I hated school, studied less and less, although I always loved gaining knowledge, and stopped communicating with my peers. Now I understand that such persistent study of the French language allowed me to eventually enroll in a bachelor’s degree at the Faculty of Philology of *** State University without exams (though I didn’t really like it there, to put it mildly), and then study for a master’s degree and work in France (I really liked it there) . But I still believe that the repeated choice not to change school led to the fact that in adolescence I forgot how to study and lost any goals other than the All-Russian school Olympiads. It took a long time to become interested in the world again. Now I am still perplexed why my mother made such a decision, although in the 70s she herself graduated from one of the leading competitive schools in ***, but I guess that she wanted a future that was understandable to me without the miracles that happen in schools where they enter through a competition. |  | not changing school | education, parent-child relationships, bullying |  |  |  |  | In 2018, I decided to go to study for a master’s degree in France, despite the fact that there was no one to expect money or help from - then this project seemed crazy. It was scary, especially since I entered the “stage art” program - complete uncertainty. Now I am very glad that I made this decision. Then it seemed to me that I was embarking on a crazy, not entirely justified adventure. Now I understand that it was a great decision, which opened up many paths of development for me and helped me find what I want to do, that is, it determined my future path. | higher education choice | moving abroad, education, money |
| 22 | A strange situation happened to me.  On 05/14/2022 at 20:14 I uploaded it to the Smart LMS platform. Due to the fact that the deadline for its mandatory uploading was agreed upon by 24.00, I took a photo of the uploaded review just in case, so that there would be objective reasons to prove that I uploaded the review within the established time frame. On June 02, I saw the review score and was surprised, because I approached the preparation of the review carefully, did a qualitative analysis of the article, and wrote a meaningful review. I myself am the author of a number of publications, including in peer-reviewed journals, and have some experience working and communicating with reviewers of journals of various statuses. I immediately wrote letters to the head of the program, *** “Mago Le-go” “Terra Incognita of modern childhood” and the teacher of the program *** I was pleased with the quick response from the teachers 06/03/2022  *** She explained to me the reason for the 6-point rating of the review due to the fact that 2 columns of the table were not filled in. However, when I turned to the photo of the screen with the loaded review, taken on May 14, 2022 at 20:14, I was very surprised that I was missing exactly those columns that were included in the photo, which I immediately wrote to *** in a response letter *** with the photo sent. *** asked me to send a review, and I sent it to her, but first opened the file with it on my desktop. *** in her next letter clarifies why the deadline for saving the review is 06/03/2022, and I explain to her, since I have the auto-save option in the system on my computer.  Every time I open a file on my computer, it automatically displays the date it was opened, so the date it was opened was recorded, not the date it was sent. However, when opening a link from a specialist, the contacts that were indicated to us in classes in case of contacting him for help and clarification were given to ***. I was shocked because my original table from May 14 contained text in all columns according to the criteria. I asked to reconsider my grade, but it was not revised and the points were not raised. I am frustrated and saddened by this situation. |  | handing in wrong assignment | education, teacher-student relationships, technologies |  |  |  |  | I graduated from the Art and Technology Lyceum, I always drew,  worked with clay with passion, I have a great sense of space and texture. I think these artistic skills help me observe people, subtly grasp the nuances of a person’s psychological portrait, anticipate people’s behavior, and establish communications. In high school, he studied enthusiastically, was interested in history, literature, geography, foreign languages, attended many clubs and sports sections: ballroom dancing, karate, chess, vocals, and graduated from a music school in classical guitar. The question of choosing a higher education was not a question for me; the clear decision was to be a psychologist. I began my studies at the *** State University of Psychology and Education with prominent representatives of Russian psychology: E.I. Isaeva, I.Yu. Kulagina, T.V. Basilova, V.V. Rubtsov and others. These were unforgettable eventful meetings for communication and professional growth.  While studying at the *** State University of Psychology and Education, I gained extensive experience as a psychologist - a practice  that I already use in my practical work, and I develop and grow in the process of live dialogue and polylogue, just like L.S. Vygotsky, when he describes the zone of proximal development of a child with an intelligent adult who truly develops simply because he is. Took part in the international competition named after L.S. Vygotsky this year, unfortunately, did not win a prize, but he performed, prepared adequately, wrote a poem, made an arrangement, and recorded a video. I am an active student focused on both process and results, and most importantly, I am deeply interested in counseling psychology. When writing notes for classes and trainings in practice, I used fundamental theses about personal growth, the search for the meaning of life, and constructive personal development. I began to show my interest in personology and counseling psychology in my 3rd year, when I first became acquainted with the books of K. Rogers “Client-Centered Therapy”, I. Yalom “Existential  Psychotherapy”, E. Berne “Transactional Analysis and Psychotherapy”. The admission procedure itself was a significant personal event for me. The HSE master's program in personology and counseling psychology will help. In the future, I wanted to become  a certified consultant, since in an age of uncertainty, the number of clients in need of quality consulting is increasing. I strive to approach the client’s difficulties professionally and with sensitivity in order to help everyone realize their potential and change their lives for the better. Studying at HSE has always been a dream for me, not only because I watched many lectures by HSE professors and associate professors online. I understood that I was being taught some completely different psychology, which fascinated me and opened up life prospects and priorities in a different way. Each meeting with HSE teachers, both online and in person, has an eventful, significant status. As a result of meetings (lectures, viewing presentations at conferences, publications in scientific literature, your  website, we were with you - the direction of movement, the general picture of my life, the research being carried out suddenly became so clear and tangible, and I finally began to understand in which direction move on, important discoveries and revelations thanks to you and the necessary steps. New meanings became clear in my research, it deepened, became more refined with other content. This brought relief and a sense of security. | higher education choice | education, art |
| 23 | A situation that I consider a bad choice is untimely and thoughtless admission to a master's program. The decision to enroll was made in July 2021.  Before explaining this choice, it is necessary to clarify some details of the relationships in my family. The fact is that the largest and most influential part of my family expected me to enroll in any, in their opinion, prestigious master's degree program, as long as I got the credentials. I didn’t mind, but I wanted to work, relax after my bachelor’s degree, and think carefully about this step. This position was contrary to the position of the family - they wanted me to go to a master's program immediately after a bachelor's degree.  Unfortunately, relationships in my family have developed in such a way that I am very afraid of disappointing my parents and loved ones, I always strive to earn their love and respect. Due to the desire to meet their expectations, I made the following decision: if I enter a master’s program that I like on a budget, I will go, if I don’t enter, I will work. I entered on a budget in a field that I like. I did not feel joy, a burden of responsibility fell on me, and I knew that it would be difficult for me to combine work and study. I knew I was tired of doing science. I knew that my body needed a break. I knew I wouldn't be able to devote enough time to my career. However, I followed my own fear of disappointing my parents and decided to study. My expectations were largely met. Now I’m finishing the first year of my master’s degree, I like many subjects, but I’m absolutely emotionally burnt out from combining a heavy workload and study, and I’ve practically stopped enjoying both the first and the second. Now I feel regret and resentment. Of course, I also see advantages in this situation: I learned a lot of interesting things, became close to wonderful people, learned a lot, moreover, I will probably use the knowledge I received. However, if I had the chance to change the past, I would delay enrolling for at least a year, and, more importantly, let my family know that I am an independent adult capable of making these decisions on my own. |  | higher education choice | education, parent-child relationships, health, burnout, work |  |  |  |  | In this essay I would like to talk about a situation that happened to me in August 2021. When I graduated from my linguistic bachelor's degree in June 2021, I was faced with the question of choosing a career path. For me, the choice was obvious: I always wanted to be a teacher, but I was a little worried about the lack of pedagogical education. As a result, in July 2021 I went to work in a kindergarten, I liked the work, the salary suited me, and no pedagogical education was required. I had just gotten used to the role of a teacher, got used to the regime and the children, when suddenly I received a job offer from an old girlfriend, with whom we studied together at one of the *** schools. She is a little older than me, so she had already worked as a teacher for two years at the school where we both graduated, and she invited me to become colleagues.  I was confused. On the one hand, it was my dream job. My favorite school, working with children, potential experience - everything pushed me towards a positive answer. And yet there was one “but”: what if I can’t cope? In kindergarten I had enough enthusiasm and love for children, I worked in a small nursery group. I understood and felt that school required a completely different level of preparation. I delayed the moment of making a decision, shifting responsibility for this decision to other people. I consulted with friends and family, weighed the pros and cons for a long time, but in the end I decided to take a risk and accept my friend’s offer. I went to work as a teacher in a secondary school. I was incredibly lucky with the children I had the opportunity to teach, with the team I got to join. I gained invaluable experience this year and do not regret my decision at all. Now I am grateful to myself for believing in myself. It was hard, I struggled every day with the impostor syndrome, which was fueled by the words of the children’s parents, who did not believe that the young teacher could teach anything. But every time I see the sparkling eyes of a child running to my lesson, I feel that I did everything right. | work choice | friendship, work, interpersonal relationships, relationships with colleagues |
| 24 | My unfortunate case was entering a master's program at the Higher School of Economics, which did not suit me. At that time, I had already mastered one psychological approach and wanted to get a higher psychological education (without a big difference in the approach, because I was going to work in the original approach). Admission took place in the summer of 2020, in October 2020 I began studying in ***, and within a month I realized that the program I had chosen was not suitable for me and that I did not want to continue studying in it. The main participant in this situation was most likely only me, since it concerned exclusively me and my self-determination. It is difficult for me to identify any other participants in this situation, except for those who surrounded me and were nearby, but did not take direct part in the situation (my family, teachers at the university). The feelings at that time were very difficult: a feeling of failure, fears that something was wrong with me, since I have difficulties with learning and I do not like such a popular and advertised program. In addition, all my friends and relatives knew that I was going to change my profession and study to become a psychologist, and it was not easy for me to fail in this way. Plus, I blamed myself to some extent for not familiarizing myself with the content of the program in more detail and not preparing for admission from this point of view. At that moment, despite the fact that studying in this program would be easier for me than in another (more loyal forms of control, the opportunity not to master academic disciplines that are difficult for me, the opportunity to obtain a diploma of higher psychological education as quickly as possible and start my consulting practice), I decided to drop out and enroll next year in a more difficult master’s program for me. Over the course of a year, I prepared, studied a lot of literature on the topic, and ended up in a program that I really like, and on studying which I don’t mind spending time, effort, money, and most importantly, I see the meaning and confirmation of my values, because which I left. This year after expulsion was filled with different experiences for me - that I had failed, that I might not get in at all, that I might not like it again, that it would be difficult to combine full-time full-time studies with 2 children. Now I understand that this was the best choice for me and this situation allowed me to make a real choice in favor of myself, albeit a more difficult one, rather than endure and wait out not very successful circumstances. Now my feelings in connection with this situation have changed, now I feel the value of the experience gained, the importance of trusting myself, believing in myself and the ability to determine life on my own, change it, make mistakes. All this is ok. |  | higher education choice | education, relationships with children, work |  |  |  |  | As I already said in the previous essay, after failure, I entered another MP at the HSE - counseling psychology, and in this program I really like the training and content with disciplines, despite the fact that studying can be difficult. Admission took place in the summer of 2021, the main participant, again, was myself + my family, who supported me during this time and gave me the opportunity to study, + members of the admissions committee, so to speak. Even at the stage of conversation with the commission, I felt “at home”, realized the correctness of my current choice and that it was not in vain that I had worked for almost a year to achieve this goal. Now this situation evokes a feeling of gratitude, self-confidence, desire to learn and develop further. I am very happy with my choice, it has significantly expanded my social circle and allowed me to develop and gain new competencies, as well as start working in this field. If we talk about feelings then, I couldn’t believe that all this had finally happened, and that now everything would be different. Now I think a lot about how self-support + personal therapy was very important for me, and also about the fact that before making important and life-shaping decisions, you need to carefully imagine how these decisions in detail can affect your life. I felt like I didn't have enough of this before. | higher education choice | education, relationships with family, teacher-student relationships, work |
| 25 | After finishing my bachelor’s degree - in 2019, in *** - I was faced with a choice: go to study at an “old” university with friends with familiar teachers or go to a new, even more high-status university with a completely different approach to education, that is, as I thought, start everything from scratch. Based on my scores, I got into both universities. I couldn’t make a decision for a long, long time - in the end I chose the second option. A year later, I took the documents back, being in deep depression.  I interviewed all my acquaintances, friends and relatives in search of the “correct” answer. I didn’t understand at all what to do - I couldn’t hear myself. At the same time, I clearly understood that studying at a new university would predetermine my life for several years to come, and that my well-being and inner comfort in the coming years depended on this choice. This is probably why it was very scary to make a decision and make a mistake.  Now I understand that I took the wrong step. On the other hand, then I couldn’t even imagine what awaited me: I studied in a non-core (for a university) direction, the subjects in which were taught disgustingly; the learning process was not built in; There were 2 scientific supervisors per 100 students, etc. And, of course, it’s a great pity that I can safely erase one or two years from my life - two very valuable years: I was very worried about the “unsuccessful” master’s studies, which affected other areas of my life during that period. The most The unpleasant thing is that now I (seemingly) understand that I intuitively knew that I needed to stay at the “old” university, but for some reason I made a different choice. Why? - I don't know. |  | higher education choice | education, interpersonal relationships, parent-child relationships, relationships with peers, heatlh |  |  |  |  | In the summer of 2021, I decided to move to ***.  I felt out of place in my hometown, so I decided to “change the situation.” The decision was made in a matter of days - and I perfectly understood that this was a crazy decision: I had a wonderful job, colleagues, home, family and a little internal discomfort. Based on this feeling, I decided to move. The discomfort was due to the fact that after 5.5 years of living in the northern capital, my hometown seemed boring and uninteresting to me: different people and a different atmosphere. It seemed to me that life was passing me by. *** attracted me with its energy and drive. Having followed the already trodden path, I consulted with everyone close to me, who for the most part supported me.  I entered HSE in a non-core (for myself) direction, which, by the way, I have never regretted. It was very, very scary when moving - I had no idea what awaited me. But I didn’t want to stay either.  Now I understand that it was a good choice. But at the same time, I understand perfectly well that everything could have turned out differently - both then (in 2019) and now (in 2022). It seems to me that there is no such thing as a good or bad choice. After all, when we make this or that decision, we rely on our feelings at that particular moment. It seems to us that this is the best solution - that we cannot do otherwise. And after everything works out (and everything can work out differently), we evaluate our decision. What determine the consequences of our choice? I think that mostly from unforeseen circumstances that we cannot control. | moving to another city | education, relationships with family, teacher-student relationships, work, interpersonal relationships |
| 26 | I consider the choice of profession as an auditor to be my poor choice in life. At that period of my life, in 2000, I set a goal to become successful and independent, to build a career. To do this, at the age of 17, I needed to leave my hometown for a bigger city. My parents are both doctors, and I always had a desire to help people. I thought that I could become a good surgeon, cardiologist, or resuscitator, but I was sure that it was impossible to achieve a high financial position by working in this field. My parents unobtrusively encouraged me to enter a medical school, they said that it would be easy for me to study, I had the ability, but I fundamentally went against their opinion, I rebelled, because... and relations with them at that time were tense. I was sure that I was right and did not doubt it at all. I can’t say anything specific about my feelings then, or even now, the only thing is that now I would change my mind and sometimes I often regret that I am not a doctor. |  | higher education choice | education, parent-child relationships |  |  |  |  | Of course, I consider the decision to retrain as a psychologist to be an important choice in my life. For a long time, after 30 years, I believed that it was too late to change anything, that time was lost, but I continued to read books on psychology in different directions, watch interviews, read articles, and take part in trainings. One day I decided to try and experience for myself what psychotherapy is and went to my first session with a psychologist. This event turned my mind upside down, and I instantly realized that this is what I want to do, this is where I will realize my unfulfilled dream of helping people. I can’t say that it’s easy for me now, it’s a colossal amount of work, because now I have to combine studying with work and family, my personal unworked out moments came up and I went to therapy again, but it brings me a lot of pleasure, I’m completely satisfied with myself and confident that I am on the right path, which gives my life meaning. | higher education choice | education, psychotherapy, work, relationships with family |
| 27 | Five years ago I received a marriage proposal. The events took place in my homeland in ***. I didn’t like the young man, but I respected him and was grateful for his good attitude towards me and a pleasant pastime. I was flattered that I received a marriage proposal so early. Moreover, the candidate was very worthy and if I married him, I wouldn’t have to worry about my future. At that moment, I was pleased with myself, happy about the opportunity to choose and the prospect of successfully getting married. I didn’t think much about this topic, I just enjoyed the moment. When the time came to go to university, I unexpectedly chose a university in another country and, without thinking, went to study in ***, simply presenting the young man with a fait accompli after buying plane tickets. I didn’t care at all what he would think or worry about, I did what I wanted at that moment. At the moment, this situation makes me a little sad. There is no sense of missed opportunity, but I sometimes think about how my life would have turned out if I had married that young man. Nowadays my personal life is not easy and in moments when I feel especially lonely, I remember this opportunity to get married, which I myself so playfully refused. |  | not agreeing to marriage proposal | education, romantic relationship, moving to another country |  |  |  |  | When I was a second-year undergraduate student, I very unexpectedly lost the maximum discount on tuition at HSE due to a retake. This was a blow for me; I understood that there was no way to pay for such an expensive education. Moreover, I came from another country, where the difference in salaries and currency with *** is noticeable. The pressure was enormous, so I started seeing a psychologist. I began to consider options about returning home, transferring to another university, changing the study program. All these thoughts simply caused horror; I didn’t know what would be the right thing to do in this situation. A choice arose: transfer to another university or continue studying at HSE. I chose to stay at HSE and compete. It was very difficult, but the result exceeded all expectations. I very quickly transferred to a budget program, successfully completed my bachelor’s degree and even entered a master’s program on a budget basis. Remembering that situation, I am very proud of myself, I am filled with a feeling of joy that I did not give up on my dream during that difficult period and made the absolutely right choice. I'm happy that I made the right decision. Of course, those difficulties were worth the result. | not quitting higher education | education, psychotherapy, work, money |
| 28 | I could write that I consider enrolling in the physical education program (bachelor’s degree, Russian State University of Physical Education) to be a bad choice, but this is not entirely true. Rather, it was a necessary choice (that’s how I thought of it then, and that’s how I still think of it now). It was not the most pleasant experience, the university was not as worthy as it was described. However, the experience is necessary for the profession in which I work now. Of course, today I would choose a different program, which is why I am studying for a master's degree. During my undergraduate studies, I felt cheated. The knowledge I received was seriously different from my expectations. Today I look at this situation differently. I’ve found a lot of things useful in life and work, and I’m trying to work out the rest on my own. |  | higher education choice | education, work |  |  |  |  | Enrolling in the master's program in literary arts at the Higher School of Economics was a good choice. Compared to my previous answer, my expectations were more than met. For a year and a half I lived in anticipation, tried to enroll twice, and was very glad that I passed on a budget. Today, studying in the program evokes only joyful emotions. Everything expected happened. There are more opportunities. | higher education choice | education |
| 29 | My biggest bad choice was that when I broke up with my boyfriend, I chose the “humiliated” route. 3 years have passed and I still can’t understand why I didn’t respect myself so much. This happened in ***, but to be more precise, it all happened in the immanent essence of my soul. Then I was 21 years old, I loved the guy very much, just like he loved me. Everything was fine with us. Even now, after breaking up, we are like family, sometimes we live together, we always help each other. This is how our families always interact. But then, when we broke up, for some reason I forced myself to accept insults directed at me. Everyone told me to stop humiliating myself like that. Everyone respected and respects me, but then I assessed myself at the level of dirt. I kept trying to talk to the person, trying to get the relationship back, but my ex-boyfriend was having a great time with others. I was so hurt by this, I knew that his ex-boyfriends were also discussing me then, they knew. Instead of telling yourself: “Enough is enough. Know your worth. You didn’t find yourself in a trash heap.” I told myself: “Humiliate yourself, endure it all, do everything that this person says.” Then I understood that there were moments when I was being manipulated, or rather my feelings, but I was still obsessed with a person and completely forgot that I had a mind, consciousness, values, etc. I was even ready to meet some strange guys to make him jealous. And now I sit and think what an idiot I was. How did I allow myself to behave like this? I was raised in a family of teachers, professors. I was always taught to be strong and handsome, but instead I broke it all for this man. Even in my bachelor's degree, I have the most unusual growth chart within the quality of education in the form of the letter V. When from 100% I fell to 0 (separation), but then I again made myself who I am.   When I was all depressed, running like a dog to this guy’s every call, I said to myself: “Enough!” I changed my hair color to completely ashy. I value my hair very much, but having radically changed the color from dark blond to ashy, I said that this was the birth of a new Dima. I went into the hall so that from that weak guy I could look at the strong Dima. I began to study languages intensively, began to prepare for competitions and conferences. As a result, I became student of the year, won international conferences, received many connections, and the coolest thing is that I received an invitation from my dream university, HSE. No one could understand how I changed so much, but I changed because of this choice. The paradox of life. I hate that choice, it killed everything in me, but at the same time, thanks to this choice, I became a better version of myself. Of course, even now I want to return those kind and naive qualities that were in me before and I’m working on it. Because sometimes I scare myself. I have become too strong, many people feel pressured, but in reality I am very kind. Therefore, sometimes this choice is like some kind of cage from which I can’t get out. Everything is fine with me, I have a boyfriend, I also communicate well with my ex and he is like a brother to me, but still at times I go back to that past, look at old correspondence, photos with this ex and say to myself: “What a pity, that we couldn't be together. You yourself are rejecting me. Why can't we be together when we complement each other perfectly?" One thing I know is that now I won’t let anyone break me like that again. More precisely, he is not so guilty, it was I who humiliated myself. Therefore, in any situation you need to be strong and know your worth with the understanding that you are priceless. |  | quitting romantic relationship | romantic relationship, education, work, loneliness, moving, parent-child relationships, relationships with friends, lgbt |  |  |  |  | Good choice.  My lucky choice was meeting that ex 3 years ago when I turned 21. Because with him I began not only to exist, but to live and enjoy life. Everything was in ***. This man opened my eyes to things that I considered petty. My first serious love, first sex, meeting very interesting people. I finally accepted my sexuality, I saw cities, I learned to plan my life. My whole life was filled with new colors. You know, not everyone will approach me, because I am always cautious and perhaps this model of life did not allow me to live brightly and beautifully. He taught me to live without stereotypes and boundaries. Therefore, I have always appreciated, appreciate and will appreciate this person. Therefore, even after so many years, we are all the same family as before. This was my best choice, because if it weren’t for this person, I would not have won conferences, competitions, or studied where I wanted. Because deep down in my heart I never wanted to give in to him. He is one of the best graphic designer in the world among rank 7 college students around the world. Therefore, I had no right to be worse than him. It was with him that I discovered the potential that was in me. I would dream of returning it all at the level of love within a relationship, but life dictates its own rules. Now I have a different person and I am also happy. And that choice is always a great choice. I repeat, if I had not chosen this person, then I would have been just Dima, who could not reveal his full potential. | romantic relationship continuation | education, romantic relationship, work, lgbt |
| 30 | In 2013-2015, when I was 13-15 years old respectively and I was in grades 8-10 at school, I was in a very difficult and unpleasant romantic relationship with my classmate P. We were good friends for about a year before that, I helped P ... even in a relationship with another girl, L., who later became his girlfriend. The problem with the situation was that P. and I began to have a romantic relationship at the same time, and I really regret this choice. I didn’t have enough courage and experience all this time to confess to L. what was happening behind her back and to talk through all my feelings with P. I constantly felt like a bad person, stupid and powerless, weak and cowardly, because I couldn’t find have the courage to talk about this situation and end it. In parallel with this, I felt that I was not good enough, beautiful, or kind enough for P. to choose me for a relationship, not L, of the two of us, although I felt “special”, a kind of secret and secret. Now I only feel sympathy for myself at that age, that there were no adults around whom I could trust, or friends who would really give me strength and determination. I don’t really regret P. and L., although to be honest I’m still interested in asking how they are doing through friends, but rather for selfish reasons, they are still together in an abusive relationship, I’m glad that I still found the strength then, even after 2 years, and isolated myself from this situation completely. I’m even partly proud of myself that I got out of this situation without too many losses and became stronger. |  | starting romantic relationship | romantic relationship, interpersonal relationships, relationships with peers, education |  |  |  |  | From recent. On June 13, 2022, a friend of K. posted on social networks a question whether anyone would like to join her for a trip to Pyatigorsk on June 15 for 3 days. My fiancé invited me to go and offered to pay for the trip, since I myself did not have any extra money due to a change of job and this was my argument against such an offer. I agreed to go at his expense, wrote to my friend K. and on the same day we bought tickets, rented accommodation, and went on a plane early on June 15th in the morning. I consider this a good decision, because I did not focus on my fears and doubts, in a short time I made sure that I was taking everything important and necessary with me, I took a responsible approach to planning and everything turned out great. We got to know K. well, whom we had only met once before, saw beautiful landscapes, and although at night I had to do a test assignment for a new job, and half an hour before departure take a Magolego exam at the airport, I didn’t care I had no regrets either before or after the trip. It was also an important and successful decision for me, because I have been undergoing therapy for GAD for almost a year, now I am finishing it, which mainly manifested itself in travel and public transport, and I am very proud of myself and my body that we were able to carry out this sudden journey together . Now I feel incredibly free, as if I can do anything in the world and as if there is nothing more important than the moment of life itself. | travel l ing | psychotherapy, romantic relationship, money, travelling, work, interpersonal relationships |

**Table 3**

*Participant list*

| **ID** | **Gender** | **Возраст** |
| --- | --- | --- |
| 1 | female | 26 |
| 2 | female | 25 |
| 3 | female | 22 |
| 4 | female | 25 |
| 5 | male | 24 |
| 6 | male | 24 |
| 7 | male | 23 |
| 8 | female | 22 |
| 9 | female | 32 |
| 10 | female | 24 |
| 11 | female | 23 |
| 12 | female | 23 |
| 13 | female | 23 |
| 14 | female | 22 |
| 15 | female | 27 |
| 16 | female | 22 |
| 17 | male | 33 |
| 18 | female | 22 |
| 19 | female | 23 |
| 20 | female | 22 |
| 21 | female | 24 |
| 22 | male | 23 |
| 23 | female | 21 |
| 24 | female | 36 |
| 25 | female | 24 |
| 26 | female | 39 |
| 27 | female | 23 |
| 28 | female | 23 |
| 29 | male | 24 |
| 30 | female | 21 |

# Survey questions, regarding life choices in Russian

Пожалуйста, вспомните о двух событиях в Вашей жизни и опишите их.

*Первое событие*

Расскажите в формате эссе 300–1000 слов о любом случае из Вашей жизни, который Вы рассматриваете как НЕУДАЧНЫЙ выбор, существенно повлиявший на Вашу жизнь сейчас.

При повествовании сфокусируйтесь на следующих моментах:

-что произошло (суть ситуации);

-когда это произошло (время);

-кто входил в число участников данной ситуации (участники);

- где это происходило (место);

-какие чувства тогда вызвала эта ситуация (чувства тогда);

-какие чувства сейчас вызывает у Вас эта ситуация (чувства сейчас);

-какие мысли сейчас вызывает у Вас эта ситуация тогда (мысли тогда);

-что Вы думаете о ситуации сейчас (мысли сейчас).

*Второе событие*

Вспомните, пожалуйста, и опишите в формате эссе 300–1000 слов о любом случае из Вашей жизни, который Вы рассматриваете как УДАЧНЫЙ выбор, существенно повлиявший на Вашу жизнь сейчас.

При рассказе сфокусируйтесь на следующих моментах:

-что произошло (суть ситуации);

-когда это произошло (время);

-кто входил в число участников данной ситуации (участники);

- где это происходило (место);

-какие чувства тогда вызвала эта ситуация (чувства тогда);

-какие чувства сейчас вызывает у Вас эта ситуация (чувства сейчас);

-какие мысли сейчас вызывает у Вас эта ситуация тогда (мысли тогда);

-что Вы думаете о ситуации сейчас (мысли сейчас).

# Survey questions, regarding life choices in English

*First Event*

Tell, in essay format, in 300-1000 words, about any event in your life that you view as an UNFORTUNATE choice that has significantly affected your life now.

When narrating, focus on the following points:

-what happened (the nature of the situation);

-when it happened (time);

-who was involved in the situation (participants);

- where it happened (location);

-what feelings the situation evoked for you then (feelings then);

-what feelings this situation is causing you now (feelings now);

-what thoughts you have now about the situation then (thoughts then);

-what you think about the situation now (thoughts now).

*Second Event*

Tell, in essay format, in 300-1000 words, about any event in your life that you view as an FORTUNATE choice that has significantly affected your life now.

When narrating, focus on the following points:

-what happened (the nature of the situation);

-when it happened (time);

-who was involved in the situation (participants);

- where it happened (location);

-what feelings the situation evoked for you then (feelings then);

-what feelings this situation is causing you now (feelings now);

-what thoughts you have now about the situation then (thoughts then);

-what you think about the situation now (thoughts now).

# Ethical statement

Informed consent was signed by study participants. Participants' personal data was not included in the analysis. The data was only being used by the research team. The study does not go against the ethical guidelines set forth by HSE University (<https://www.hse.ru/en/org/hse/irb/ethics>).

# Examples and suggestions for writing exercises related to career counseling.

## Recommendations and examples for teachers use:

Lengelle, R., & Ashby, S. (2017). Writing as soul work: training teachers in a group-based career-narrative approach. British journal of guidance & counselling, 45(4), 402-416. doi:10.1080/03069885.2016.1169366

## Recommendations and examples for counsellors use:

da Silva, C. S. C., Teixeira, M. A. P., Cardoso, P., Fernandez-Navarro, P., Gonçalves, M. M., & Duarte, M. E. (2020). Innovative moments and narrative change in career counselling: a case study. International Journal for Educational and Vocational Guidance, 20, 635-652. doi: 10.1007/s10775-020-09422-7

Cardoso, P. M., Savickas, M. L., & Gonçalves, M. M. (2021). Facilitating narrative change in career construction counseling. Journal of Career Development, 48(6), 863-876. doi: https://doi.org/10.1177/0894845319898872

## Two-day writing course for counsellors and teachers use:

Lengelle, R., Meijers, F., Poell, R., Geijsel, F., & Post, M. (2016). Career writing as a dialogue about work experience: A recipe for luck readiness? International Journal for Educational and Vocational Guidance, 16, 29-43. doi: 10.1007/s10775-014-9283-1

Lengelle, R., Meijers, F., Poell, R., & Post, M. (2014). Career writing: Creative, expressive and reflective approaches to narrative identity formation in students in higher education. Journal of Vocational Behavior, 85(1), 75-84. doi: 10.1016/j.jvb.2014.05.001

## Full-length manual of narrative career counselling for proffessuonal counsellors:

Maree, K. (2019). Shaping the story: A guide to facilitating narrative career counselling. BRILL.

1. Translated from Russian. [↑](#footnote-ref-1)
